# Inhibition of phosphodiesterase-4 promotes oligodendrocyte precursor cell differentiation and enhances CNS remyelination

**DOI:** 10.1002/emmm.201303123

**Published:** 2013-10-21

**Authors:** Yasir A Syed, Alexandra Baer, Matthias P Hofer, Ginez A González, Jon Rundle, Szymon Myrta, Jeffrey K Huang, Chao Zhao, Moritz J Rossner, Matthew W B Trotter, Gert Lubec, Robin J M Franklin, Mark R Kotter

**Affiliations:** 1 Wellcome Trust and MRC Cambridge Stem Cell Institute, and Anne McLaren Laboratory for Regenerative Medicine, University of CambridgeWest Forvie Building, Forvie Site, Robinson Way, Cambridge, UK; 2 Department of Neurosurgery, Medical University ViennaVienna, Austria; 3 Max-Planck Institute for Experimental Medicine, Department of NeurogeneticsGoettingen, Germany; 4 Department of Pediatrics, Medical University ViennaVienna, Austria; 5 Wellcome Trust and MRC Cambridge Stem Cell Institute, and Department of Veterinary Medicine, University of Cambridge, Madingley RoadCambridge, UK; 6 Max-Planck-Institute for Experimental Medicine, Research Group ‘Gene Expression and Signalling’, Department of NeurogeneticsGöttingen, Germany

**Keywords:** demyelination, Mapk signalling, multiple sclerosis, oligodendrocytes, remyelination

## Abstract

The increasing effectiveness of new disease-modifying drugs that suppress disease activity in multiple sclerosis has opened up opportunities for regenerative medicines that enhance remyelination and potentially slow disease progression. Although several new targets for therapeutic enhancement of remyelination have emerged, few lend themselves readily to conventional drug development. Here, we used transcription profiling to identify mitogen-activated protein kinase (Mapk) signalling as an important regulator involved in the differentiation of oligodendrocyte progenitor cells (OPCs) into oligodendrocytes. We show in tissue culture that activation of Mapk signalling by elevation of intracellular levels of cyclic adenosine monophosphate (cAMP) using administration of either dibutyryl-cAMP or inhibitors of the cAMP-hydrolysing enzyme phosphodiesterase-4 (Pde4) enhances OPC differentiation. Finally, we demonstrate that systemic delivery of a Pde4 inhibitor leads to enhanced differentiation of OPCs within focal areas of toxin-induced demyelination and a consequent acceleration of remyelination. These data reveal a novel approach to therapeutic enhancement of remyelination amenable to pharmacological intervention and hence with significant potential for translation.

## INTRODUCTION

Enhancing the regeneration of myelin sheaths in the central nervous system has been identified as an important therapeutic strategy to ameliorate the devastating consequences of persistent demyelination (Franklin & Ffrench-Constant, 2008; Kotter et al, 2011). Myelin sheaths provide the structural basis for saltatory signal conduction and provide trophic support that helps maintain axonal integrity. Thus, loss of myelin sheaths (demyelination) results acutely in a conduction block (Franklin & Ffrench-Constant, 2008; Nguyen et al, 2009) and ultimately in axonal degeneration (Bitsch et al, 2000; Nikic et al, 2011). Myelin regeneration, or remyelination, is mediated by multipotent adult stem/progenitor cells (Kotter et al, 2011; oligodendrocyte progenitor cells, OPCs) that are recruited into areas of demyelination where they engage axons, and differentiate into myelin forming oligodendrocytes (Franklin & Ffrench-Constant, 2008; Kotter et al, 2011; Zawadzka et al, 2010). In chronic demyelinating disease of several decades duration, such as multiple sclerosis (MS), remyelination frequently fails because of a declining efficiency in OPC differentiation (Chang et al, 2000; Kuhlmann et al, 2008; Wolswijk, 1998). Therefore, the search for safe drugs that are able to stimulate OPC differentiation and promote remyelination represents a major focus in clinical neurology. Some hope derives from recent findings such as the identification of leucine-rich-repeat and Ig-domain-containing 1 (LINGO-1), the wnt pathway and retinoic acid receptor gamma (RXR-γ) as potential therapeutic targets (Fancy et al, 2011; Huang et al, 2011; Mi et al, 2007).

The biological process leading to OPC differentiation likely involves a cascade of molecular events that eventually determines the generation of mature oligodendrocytes. Implicit in such a model is first, the concept that the initial triggers are fewer than the signals ultimately required for differentiation and second, the prediction that manipulation of these early triggers will have more profound consequences for differentiation than later events. Thus, to identify the earliest detectable transcriptional correlates of differentiation, we conducted a microarray analysis of gene expression in primary rat OPCs undergoing rapid morphological changes in cell culture within a few hours of purification. This revealed significant regulation of several genes associated with the Mapk signalling pathway, leading us to ask whether an increase in intracellular cyclic adenosine monophosphate (cAMP), a positive regulator of Mapk signalling, could promote OPC differentiation. We show that pharmacological inhibition of the cAMP-hydrolysing enzyme phosphodiesterase-4 (Pde4) stimulates OPC differentiation in the presence of myelin inhibitory factors likely to be present in early-stage MS lesions. Finally, we demonstrate that inhibition of Pde4 promotes CNS remyelination *in vivo*. Our results suggest a novel approach for the therapeutic enhancement of remyelination in chronic demyelinating disease, such as MS (Barkhof et al, 2010).

## RESULTS

### Transcription profiling identifies Mapk signalling as potential early regulator of OPC differentiation

To identify early transcriptional events that initiate OPC differentiation into oligodendrocytes, transcriptional profiling was performed on primary rat OPC monocultures directly after purification and following differentiation for 4 and 12 h. Differential expression between sample groups was assessed using the moderated *t*-test with *p*-value correction for multiple testing at a false discovery rate (FDR) threshold of 5% (*q* < 0.05). Among other findings this revealed that several members of the mitogen-activated protein kinase (Mapk) pathway were differentially regulated during early stages of OPC differentiation. For example, Mapk phosphatase-1 (Mkp-1/Dusp1), which dephosphorylates two key members of classical Mapk and Jnk/p38Mapk signalling pathways, Erk and p38Mapk (Fragoso et al, 2007; Kaiser et al, 2004; Sanchez-Perez et al, 1998), was found to be rapidly down-regulated within 4 h of initiation of differentiation (Supporting Information Fig 1).

Given that the Mapk pathway has been previously implicated in the differentiation of OPCs (Fragoso et al, 2007) and of other cell lineages (Nebreda & Porras, 2000; Ono & Han, 2000), we explored the regulation of members of the Mapk signalling pathway in the process of myelin regeneration *in vivo* by examining dynamic changes in expression of genes associated with this pathway using previously published transcriptome data of CNS remyelination (Huang et al, 2011). This revealed changes in several genes associated with this pathway at the stage of remyelination at which OPC differentiation is initiated (Supporting Information Fig 1).

### Phosphorylation of Erk1/2, p38Mapk and Creb1 in OPCs is impaired in presence of myelin-associated inhibitors

**Figure 1:**
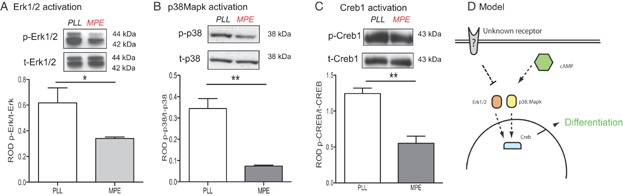
Inhibition of OPC differentiation by MAI is regulated by phosphorylation of Erk1/2, p38Mapk and Creb1.

The Mapk pathway involves both classical Erk-mediated and p38Mapk signalling. To investigate which part of Mapk signalling is primarily involved in OPC differentiation, we examined the effects of suppressing OPC differentiation on Erk and p38Mapk activation. To do this, we cultured OPCs on tissue culture plates coated with CNS myelin protein extract (MPE), which have previously been shown to contain proteins profoundly inhibitory to OPC differentiation (myelin-associated inhibitors, MAI) (Kotter et al, 2006; Robinson & Miller, 1999). This approach also has pathophysiological relevance because it is likely that uncleared myelin debris in early MS lesions contributes to remyelination failure (Kotter et al, 2011). OPCs were cultured for 24 h in differentiation medium in the presence or absence of MPE. Assessment of Erk and p38Mapk phosphorylation demonstrated a significant reduction of Erk1/2 and p38 Mapk activity in the presence of myelin associated inhibitors (Fig 1A and B).

A–C. Top, representative blots of phosphorylated (p-) protein, re-probed for total (t-) protein levels, of (A) Erk1/2, (B) p38Mapk and (C) Creb1. Bottom, relative optical-density-based (ROD) quantification of phosphorylated to total protein ratios of Erk, p38Mapk and Creb1 on control (poly-l-Lysine, PLL) substrates and in the presence of MAI (MPE). Student *t*-tests: (A) *n* = 3; *p < 0.05; PLL:0.6177 ± 0.1166; MPE: 0.3406 ± 0.01203; B) *n* = 3; **p < 0.01; PLL: 0.3438 ± 0.04692; MPE: 0.07413 ± 0.004414; (C) *n* = 4; **p < 0.01; PLL: 1.244 ± 0.07505; MPE: 0.5517 ± 0.09847.
D. Proposed model of Mapk signalling during OPC differentiation: OPC differentiation is mediated by activation of Erk1/2 and p38Mapk. Erk1/2 and p38Mapk in turn activate Creb1, which ultimately leads to OPC differentiation. The presence of MAI impairs Erk1/2 and p38Mapk activation, which results in impairment of Creb1 phosphorylation. The model predicts that an increase of intracellular cAMP results in activation of p38Mapk, Erk1/2 and Creb1 activity and ultimately promotes OPC differentiation. Error bars: SEM.

The transcription factor Creb1 is a downstream effector of p38Mapk (Foulkes et al, 1991; Krebs et al, 1997) and Erk1/2 (Esparza et al, 2008; Zubenko & Hughes, 2010). Because Mapk activation of Creb1 has been implicated in the differentiation of various cells, including OPCs (Cuadrado & Nebreda, 2010; Di Giacomo et al, 2009; Gonzalez & Montminy, 1989; Sato-Bigbee et al, 1994) we assessed Creb1 activation in differentiating OPCs. This revealed an increase in Creb1 phosphorylation associated with OPC differentiation (Supporting Information Fig 2), which was impaired in the presence of MAI (Fig 1C).

**Figure 2:**
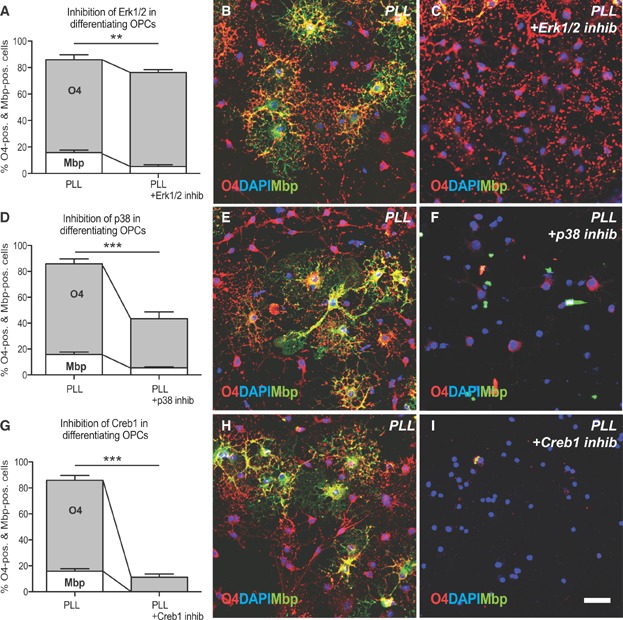
Erk1/2, p38Mapk and Creb1 play a functional role during OPC differentiation.

To functionally test the role of Erk1/2, p38Mapk and Creb1 in OPC differentiation, OPCs were cultured in the presence of pharmacological inhibitors. When Erk1/2 signalling was inhibited, differentiation was arrested at a pre-myelinating stage of the oligodendrocyte lineage in which there was a reduction in Mbp expression by O4 expressing cells (Fig 2A–C). In contrast, inhibition of p38Mapk resulted in a significant reduction of both O4 and Mbp immunoreactivity, indicating a differentiation block at earlier stages of the oligodendrocyte lineage (Fig 2D–F). The most pronounced inhibition of both O4 and Mbp expression was detected when the interaction of Creb1 with its binding partner Creb binding protein (CBP) (Delghandi et al, 2005; Weiss, 1975) was prohibited using an CBP-Creb interaction inhibitor (Whitaker et al, 2008) (Fig 2G–I).

1. Bar graph showing the decrease of Mbp-positive oligodendrocyte lineage (OL) cells after 2 days in differentiation medium in the presence of an Erk1/2 inhibitor. ANOVA: O4 *p* > 0.05, MBP **p < 0.001; Dunnett's *post hoc* test PLL versus PLL with ERK1/2 inhibitor: *p* < 0.0001; mean decrease: O4 = 11%; Mbp = 67%.
2. Bar graph demonstrating a significant decrease of O4/Mbp-positive OL cells after treatment with a p38 inhibitor. ANOVA: (O4 ***p < 0.0001, Mbp ***p < 0.0001; Dunnett's *post hoc* test PLL versus PLL with p38Mapk inhibitor: *p* < 0.0001; mean decrease: O4 = 49%, Mbp = 66%.
3. Bar graph indicating a significant decrease of O4 immunoreactivity and a complete loss of Mbp expression in the presence of a Creb inhibitor. ANOVA: O4 ***p < 0.0001, MBP ***p < 0.0001; Dunnett's *post hoc* test PLL versus PLL with Creb1 inhibitor: *p* < 0.0001; mean decrease: O4 = 87%, Mbp = 100%. Error bars: SEM.
4. Representative images of cells immunolabelled for O4 (red) and MBP (green) on control substrates and cultured in differentiation medium supplemented with ERK1/2 inhibitor. Scale bar = 30 µm.

**Figure 3:**
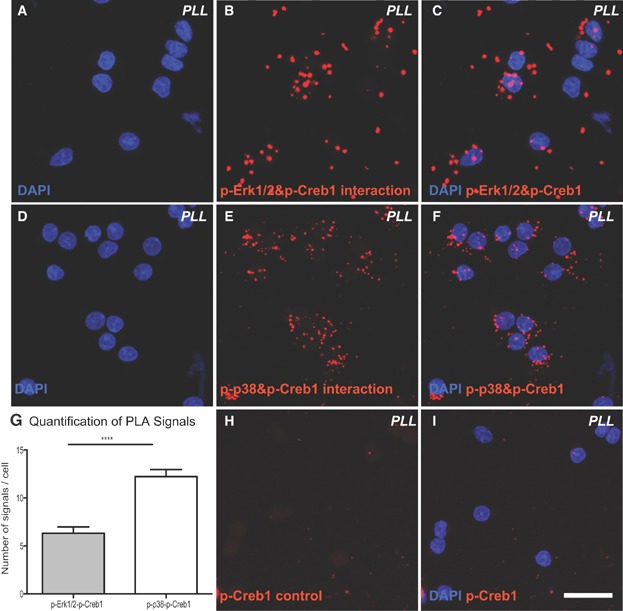
*In situ* PLA confirms direct interactions of p-Erk1/2 and p-p38Mapk with p-Creb1 during OPC differentiation. To explore interactions between phosphorylated (p-) Creb1 to (p-) Erk1/2 and (p-) p38 an *in situ* PLA was conducted. Each interaction complex detected by PLA is visualized with a fluorescent signal. DNA was counterstained by Hoechst 33342 (blue).

To investigate whether Erk1/2 and p38Mapk are directly involved in the phosphorylation of Creb1, we conducted an *in situ* protein interaction assay (proximity ligation assay, PLA). A fluorescent signal generated as a result of approximation indicated a direct interaction between of phosphorylated (p-) Erk1/2 and p-Creb1 (Fig 3A–C), and between p-p38Mapk and p-Creb1 (Fig 3D–F). Creb1 therefore seems to be directly phosphorylated by Erk1/2 and p38Mapk in differentiating OPCs.

A–C. Complexes between endogenous p-Erk1/2 and p-Creb1 were detected in the nucleoplasm and cytosol of differentiated oligodendrocytes.
D–F. Similarly, complexes between p-p38 and p-Creb during oligodendrocyte differentiation were detected.
G. Quantification of interaction complexes indicated increased interaction between p-38Mapk and p-Creb1 as compared to p-Erk1/2 and p-Creb1.
H,I. As a negative control a PLA was conducted in the absence of p-Erk1/2 or p-p38Mapk antibodies. The absence of signal demonstrates the specificity of the assay. Scale bar = 20 µm.

These findings suggest a model in which activation of Erk1/2 and p38Mapk triggers activation of Creb1 leading to OPC differentiation (Fig 1D). In contrast, MAI impair p38Mapk activation, which results in impaired Creb1 phosphorylation and hence an inhibition of differentiation. The model predicts that interventions that induce p38Mapk, Erk1/2 and Creb1 activity will promote OPC differentiation and ultimately enhance remyelination.

### Elevation of intracellular levels cAMP by dbcAMP or inhibition of phosphodiesterease-4 induces OPC differentiation in the presence of myelin-associated inhibitors

Erk1/2/p38Mapk activity can be induced by increasing levels of the intracellular second messenger cAMP. This can be achieved through at least two independent mechanisms: first, a direct interaction between cAMP and Creb1, and second by activation of the cAMP-dependent kinase Pka, which in turn phosphorylates Erk1/2 (Costes et al, 2006; Smith et al, 2010) and p38Mapk (Cuadrado & Nebreda, 2010; Delghandi et al, 2005; Gonzalez & Montminy, 1989).

**Figure 4:**
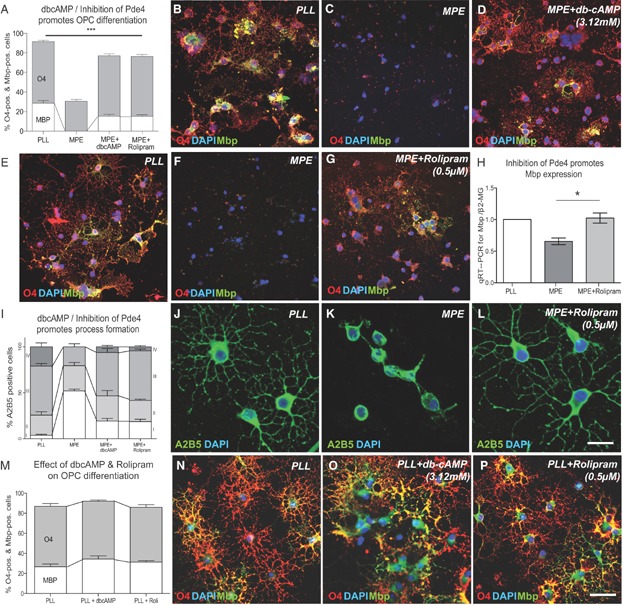
Increasing cAMP levels by treatment with dbcAMP or inhibition of Pde4 by rolipram promotes OPC differentiation in the presence of MAIs.

To test whether increasing intracellular levels of cAMP overcome the inhibitory effects of MAI, we first added dibutyryl cAMP (dbcAMP, a membrane permeable cAMP analogue) to the culture medium. Differentiation was assessed on the basis of O4 and Mbp expression, and revealed an increase in OPC differentiation after 48 h of culture (Fig 4A–D) in the presence of dbcAMP.

1. Bar graph showing the percentage of O4^+^ and Mbp^+^ OL cells after 2 days in differentiation medium plated on control substrates (PLL), MPE and MPE treated with 3.21mM dbcAMP or 0.5 µM rolipram (*n* = 3; ANOVA: O4 ***p < 0.0001, MBP ***p < 0.0001; Dunnett's *post hoc* test MPE versus MPE treated with 3.21 mM dbcAMP: *p* < 0.0001; MPE versus MPE treated with 0.5 µM rolipram *p* < 0.0001; mean increase with 3.21 mM dbcAMP = 251% for O4, ∞ for Mbp: mean increase with 0.5 µM rolipram = 248% for O4, ∞ for Mbp).
2. Representative images of cells immunolabelled for O4 (red) and Mbp (green) on PLL (B,E), on MPE (C,F) and on MPE treated with dbcAMP (D) or rolipram (G). Nuclei were visualized with Hoechst (blue).
3. Ratio of mRNA levels of myelin basic protein (Mbp) versus β2-microglobulin (β2-MG) in cells cultured on PLL, MPE and on MPE in the presence of rolipram, determined by RT-qPCR. *n* = 3; Dunnet's *post hoc* test MPE versus MPE + rolipram: *p < 0.05.
4. Morphological assessment of OPCs plated on (J) PLL, (K) MPE, (L) MPE plus 0.5 µM rolipram and dbcAMP on the basis of the following categories: I, mono/bipolar; II, primary branches; III, secondary branches; IV, membranous sheaths.
5. Bar graph and representative images indicating that treatment of OPCs cultured on PLL control substrates with dbcAMP or rolipram does not result accelerate the differentiation process. ANOVA: (O4 *p* > 0.05, Mbp *p* *>* 0.05; Dunnett's *post hoc* test PLL versus PLL + dbcAMP: *p* > 0.05; mean Increase: O4 = 7%, Mbp = 34%; PLL versus PLL + Roli: *p* > 0.05; mean Increase: O4 = 0%, Mbp = 18%. Error bars: SEM. Scale bars in B–G = 50 µm, in J–L = 30 µm, in N–P = 50 µm.

We next increased intracellular cAMP levels by inhibiting the cAMP-hydrolysing activity of Pde4 (Weiss, 1975), of which OPCs express several subtypes (Whitaker et al, 2008). Recently, a number of different Pde4 inhibitors have been used in preclinical studies (Nials et al, 2011; Schafer et al, 2010) and in clinical trials (Shih et al, 2010; Spina, 2008). To test whether small-molecule inhibitors of Pde4 can promote OPC differentiation in the presence of OPC differentiation inhibitors, Pde4 inhibitors (rolipram, milrinone, irsogladine, zaprinast and rottlerin) were added to the medium at various concentrations (Supporting Information Fig 3). All inhibitors tested significantly induced OPC differentiation in the presence of MAI. For example, the differentiation-inducing effects of rolipram, a drug initially developed as an anti-depressant, at a concentration of 0.5 µM were comparable to the effects of dbcAMP treatment (Fig 4A). Rolipram treatment resulted in an increase in the number and complexity of OPC processes, a morphological change associated with differentiation (Fig 4J–L).

An assessment of *myelin basic protein* (*Mbp*) mRNA expression using quantitative reverse transcriptase PCR (qRT-PCR) relative to *β2-microglobulin* mRNA (an internal control used for normalization) demonstrated that rolipram significantly increased the expression of *Mbp*, a marker expressed by mature myelin forming oligodendrocytes (Fig 4H). Importantly, terminal deoxynucleotidyl transferase-mediated biotinylated UTP nick end labelling (TUNEL) assays did not reveal differences in apoptosis between control and rolipram-treated OPCs plated on MPE, thus ruling out differences in cell survival (Supporting Information Fig 4). Finally, we assessed whether dbcAMP or rolipram could promote the differentiation of OPCs cultured on control substrates. No significant change in MBP expression was seen following treatment (Fig 4M–P).

### Pde4 inhibition promotes OPC differentiation by activation of Erk1/2, p38Mapk and Creb1 in presence of myelin-associated inhibitors

**Figure 5:**
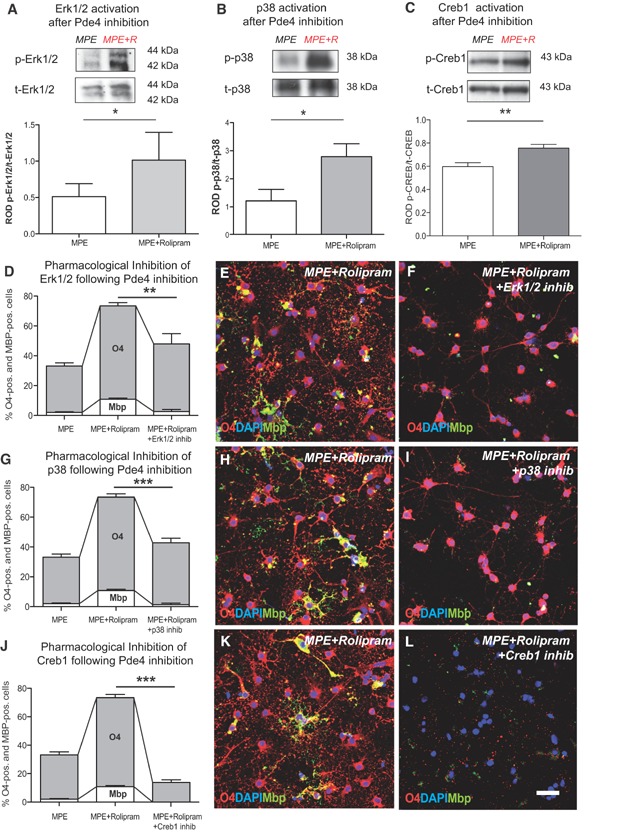
Promoting OPC differentiation by inhibition of Pde4 depends on Erk1/2, p38Mapk and Creb1 activity.

To confirm that inhibition of Pde4 acts via the proposed mechanism the phosphorylation status of Erk1/2, p38Mapk and Creb1 was assessed in OPCs cultured in the presence of MAI and treated with rolipram. This demonstrated a significant increase in the phosphorylation of Erk1/2, p38Mapk and Creb1 in rolipram-treated cells (Fig 5A–C). Furthermore, when OPCs were cultured on MAI-inhibitory substrates and treated with rolipram in combination with inhibitors of Erk1/2, p38Mapk and Creb negative effects on OPC differentiation were observed: inhibition of Erk1/2 resulted in reduced numbers of Mbp-positive cells (Fig 5D–F) and inhibition of p38Mapk in a significant reduction of both O4 and Mbp immunoreactivity (Fig 5G–I). The most extensive reduction of both O4 and Mbp expression was again detected when the interaction of Creb1 with Cbp was impaired (Fig 5J–L). These findings suggest that Erk1/2, p38Mapk and Creb1 play a role with respect for the differentiation-inducing effects of rolipram.

A–C. Assessment of Erk1/2, p38Mapk and Creb1 phosphorylation by immunoprecipitation in response to rolipram treatment. Top, representative blots; bottom, quantification based on ROD. (A) Erk1/2: *n* = 3; *t*-test: *p < 0.05; MPE: 0.3406 ± 0.01203, MPE + rolipram (MPE + R): 0.8816 ± 0.04381; (B) p38Mapk: *n* = 3; *t*-test: *p < 0.05; MPE:1.217 ± 0.4094, MPE + rolipram: 2.788 ± 0.4606; (C) Creb1: *n* = 5; *t*-test: **p < 0.001; MPE: 0.5972 ± 0.03408; MPE + R: 0.7560 ± 0.03277.
D. Bar graph showing the percentage of O4/Mbp-positive cells cultured on MPE and induced with 0.5 µM rolipram. Treatment with rolipram promotes O4 and Mbp expression but addition of a specific Erk1/2 inhibitor blocks the differentiation-inducing effects: *n* = 3; ANOVA: O4 *p* > 0.05, MBP **p < 0.001; Dunnett's *post hoc* test MPE + R versus MPE + R + ERK1/2 inhibitor: *p* < 0.001; mean decrease O4 = 35%; mean decrease Mbp = 76%.
G. Bar graph demonstrating that the percentage of O4/Mbp-positive positive cells in the presence of MPE and induced with 0.5 µM rolipram decreases following treatment with a specific pharmacological inhibitor against p38Mapk: *n* = 3; ANOVA: O4 ***p < 0.0001, MBP ***p < 0.0001; Dunnett's *post hoc* test MPE + R versus MPE + R + p38Mapk inhibitor: *p* < 0.0001; O4 mean decrease = 42%; Mbp mean decrease = 87%.
J. Bar graph indicating that the percentage of O4/Mbp-positive positive cells in the presence of MPE, induced with 0.5 µM rolipram and treated with a Creb1 inhibitor decreases below levels of cells plated on MPE. *n* = 3; ANOVA: O4***p < 0.0001, MBP ***p < 0.0001; Dunnett's *post hoc* test MPE + R versus MPE + R + Creb1 inhibitor: *p* < 0.0001, mean decrease O4 = 82%; mean decrease Mbp = 100%.
E,F,H,I,K,L. Representative images of cells immunolabelled with O4 (red) and Mbp (green). Error bars: SEM. Scale bar = 30 µm.

### OPCs express Erk1/2, p38Mapk and Creb1 in remyelinating lesions

To investigate whether OPCs express Mapk-pathway associated molecules in remyelinating lesions focal demyelination was induced in the cerebellar white matter of adult rats by the direct injection of ethidium bromide (a well-characterized model of toxin-induced demyelination thatundergoes an age-dependent stereotypical pattern of spontaneous remyelination (Woodruff & Franklin, 1999, Sim et al, 2002). Sections of lesions at 14 days post-lesion induction (dpl) were double-stained with antibodies against p-Erk1/2 or p-p38Mapk and Nkx2.2, a marker for activated OPCs (Supporting Information Fig 5A–F). This demonstrated that activated Erk1/2 and p38Mapk were present in Nkx2.2-positive OPCs. Furthermore, active Creb1 was detected in the majority of cells positive for the oligodendroglial lineage marker Olig2 within the lesions (Supporting Information Fig 5G–I).

### Inhibition of Pde4 promotes priming and differentiation of OPCs *in vivo*

**Figure 6:**
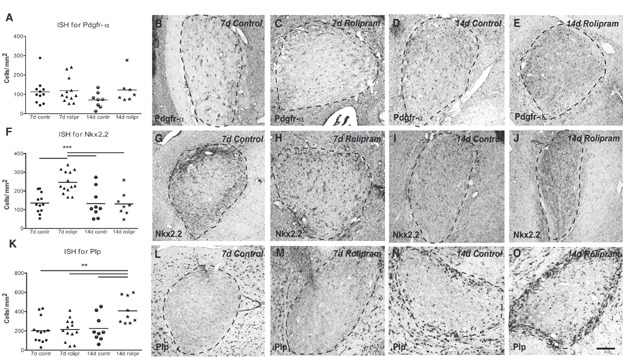
Inhibition of Pde4 primes OPCs and promotes OPC differentiation.

**Figure 7:**
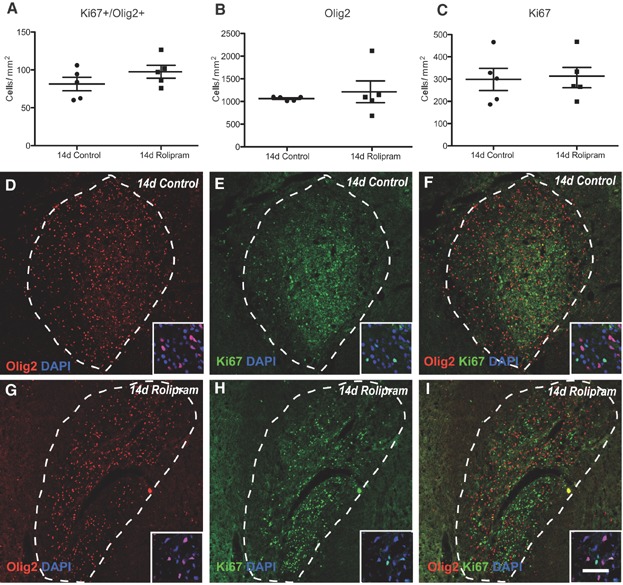
Rolipram treatment does not affect OPC proliferation in remyelinating lesions.

To assess the effects of Pde4 inhibition on remyelination, rolipram was administered via a subcutaneous minipump starting 3 days post lesion induction (dpl) and the lesions analysed at 7, 14 and 21 dpl. Assessment of the lesion size revealed no significant differences between the groups at the various time points (Supporting Information Figs 6 and 7). The expression of *Pdgfra*, an OPC marker, and *Nkx2.2*, a transcription factor associated with OPCs that are primed for differentiation was visualized by *in situ* hybridization and quantified (Fancy et al, 2004; Kotter et al, 2006). Inhibition of Pde4 affected neither the overall number of undifferentiated OPCs within the lesions as compared with controls at 7 and 14 dpl (Fig 6A–E), nor the number of Olig2^+^Ki67^+^ proliferating OPCs (Fig 7A and D–I). However, rolipram treatment significantly increased the number of *Nkx2.2*-expressing cells compared with controls at 7 dpl, indicating that inhibiting Pde4 enhances OPC priming (Fig 6F–J).

1. Scatter plot indicating that the number of *Pdgfr-α*-positive cells/mm^2^ was comparable at 7 and 14 days post lesion induction in rolipram and vehicle treated controls. ANOVA *p* > 0.1, *Pdgfr-α*: 7d control *n* = 13, 7 days rolipram *n* = 13, 14 days control *n* = 9, 14 days rolipram *n* = 9.
2. Scatter plot demonstrating a significant increase in the number of primed *Nkx2.2*-positive OPCs in rolipram treated animals compared to controls at 7d post lesion induction. *Nkx2.2*: 7 days control *n* = 12, 7 days rolipram *n* = 14, 14 days control *n* = 9, 14 days rolipram *n* = 8, ANOVA: ***p < 0.0001, Dunnett's *post hoc* test 7 days control versus 7 days rolipram ***p < 0.001.
3. Scatter plot showing a significant increase in the number of *Plp*-positive mature oligodendrocytes in the lesions of rolipram treated animals. *Plp*: 7 days control *n* = 13, 7 days rolipram *n* = 13, 14 days control *n* = 9, 14 days rolipram *n* = 9, ANOVA ***p < 0.001, Dunnett's *post hoc* test 14 days control versus 14 days rolipram **p < 0.01.
4. Representative sections labelled by *in situ* hybridization for (B–E) *Pdgfr-α*, G–J**)** *Nkx2.2*, and (L–O) *Plp* at 7 and 14 days post lesion induction. Scale bar = 200 µm.

A–C. Quantitative analysis of Ki67+ and Olig2+ cells in control and rolipram-treated lesions. Control *n* = 5, rolipram *n* = 5, Ki67 + /Olig2+ cells: *t*-test *p* < 0.05, control: 81.40 ± 8.920, rolipram: 97.57 ± 8.528, (B) Olig2+ cells: *t*-test *p* < 0.05, control: 1064 ± 19.32; rolipram: 1214 ± 239.8, (C) Ki67+ cells: *t*-test, *p* < 0.05, control: 298.5 ± 49.77, rolipram: 306.7 ± 45.50.
D–I. Representative images from control and rolipram-treated lesions at 14 dpi stained for (D,G) Olig2, (E,H) Ki67 and (F,I) Ki67 and Olig2. Scale bar = 200 µm, in insets = 50 µm.

To detect differences in the number of differentiated oligodendrocytes, cells were visualized by *in situ* hybridization for *Proteolipid protein* (*Plp*), a marker of mature oligodendrocytes (Pfeiffer et al, 1993). Rolipram treatment significantly increased the number of *Plp*-expressing cells at 14 dpl (Fig 6K–O).

### Inhibition of Pde4 enhances remyelination following experimental demyelination

**Figure 8:**
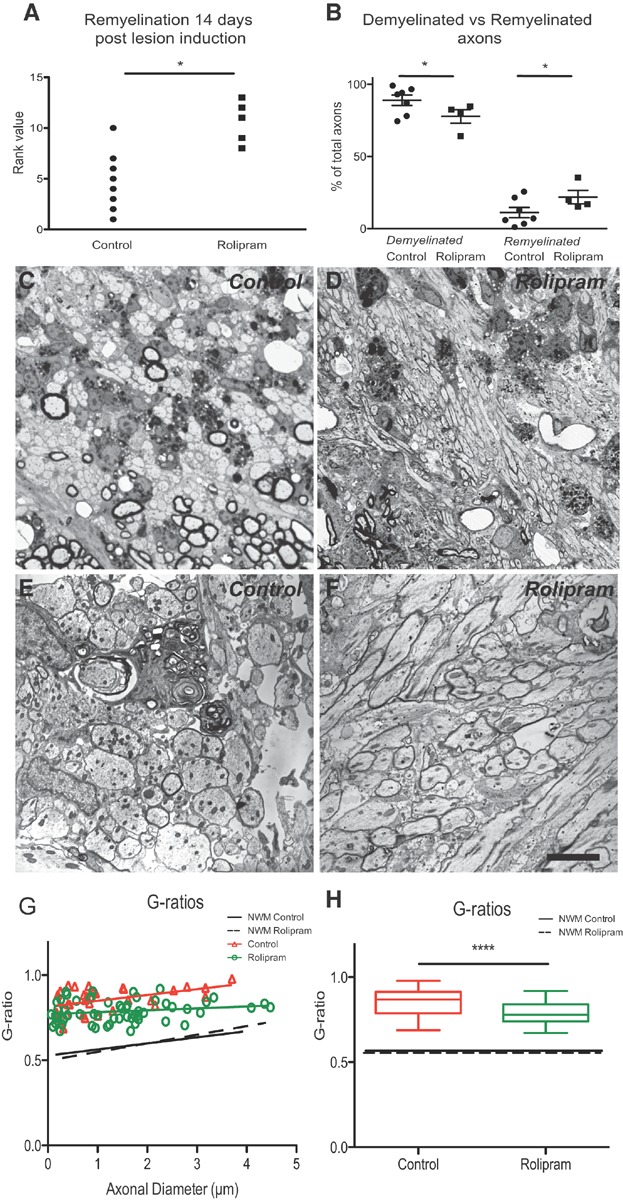
Inhibition of Pde4 promotes CNS remyelination.

In white matter tracts such as the caudal cerebellar peduncle (CCP), which contains a large proportion of large diameter axons (Woodruff & Franklin, 1999) remyelination can be reliably distinguished from myelination on the basis of thinner myelin sheaths, detectable on toluidine blue-stained semi-thin resin sections and electron micrographs (Blakemore, 1974). Whereas in control animals remyelination was restricted to a thin rim at the edge of the lesion in areas in which myelin debris was cleared, rolipram treatment increased the number of remyelinated axons around the lesion border significantly and patches of remyelination were found deeper within the lesions. Investigator-blind rank analysis of the extent of remyelination demonstrated that inhibition of Pde4 treatment significantly increased the number of remyelinated axons in the lesions (Fig 8A). These findings were confirmed by independent quantification of remyelinated axons and demyelinated axons in 1500× low power EM fields (Fig 8B). These data suggested that rolipram treatment increased the rate of remyelination, predicting that at a stage before remyelination is complete the *G*-ratio in the treated group will be lower (myelin sheath thicker). We therefore preformed a *G*-ratio analysis at 14 days (*i.e*. before remyelination is complete) and found that the group treated with the Pde-4 inhibitor had a lower *G*-ratio than the control-treated group (Fig 8G and H).

1. Rank analysis (Scatter plot) of remyelination in CCP lesions at 14 dpl assessed on toluidine-blue-stained semi-thin sections. Higher rank scores indicate greater extent of remyelination; control: *n* = 8, rolipram: *n* = 5, Mann–Whitney *U* test *p < 0.05.
2. Scatter plot representing quantification of demyelinated and remyelinated axons on electron micrographs. Control: *n* = 7, rolipram: *n* = 4, *t*-test: *p < 0.05.
3. Representative semi-thin and electron micrographs of (C,E) control and (D,F) rolipram-treated animals.
4. Scatter plot and Box plot of *G*-ratios indicating that remyelinated axons in rolipram treated animals have thicker myelin sheaths (lower *G*-ratios) than remyelinated axons in controls. (*G*-ratios of native myelin sheaths in both groups indicated by dotted lines.) Control: *n* = 34; rolipram: *n* = 58; *t*-test; ***p < 0.001; control: 0.8540 ± 0.01211; rolipram: 0.7871 ± 0.008436; Error bars: ± SEM. Scale bar C and D = 50 µm; E and F = 5 µm.

### Inhibition does not alter the innate immune response

**Figure 9:**
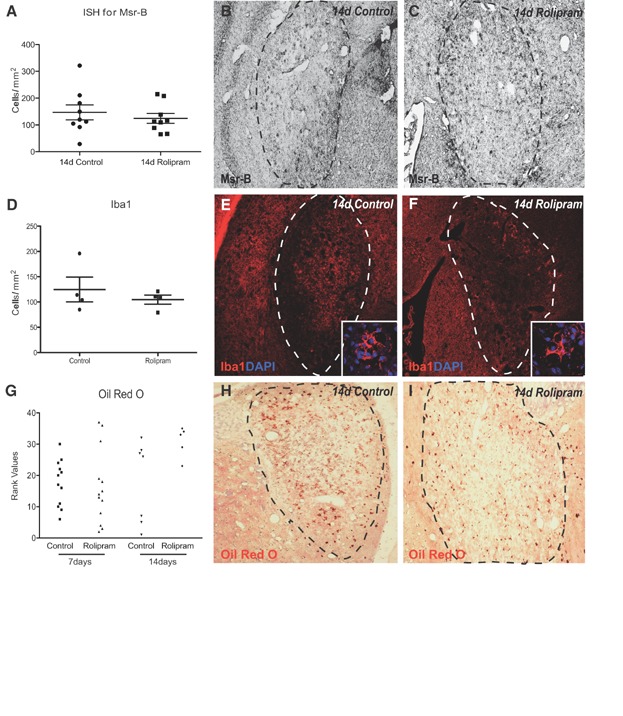
Pde4 inhibition does not alter the presence of microglia/macrophages in remyelinating lesions

The innate immune system plays an important role in myelin regeneration (Kotter et al, 2001). As rolipram can also exert effects on inflammatory processes, *albeit* at higher concentrations than the ones used in the present study (Mendes et al, 2009), the presence of macrophages within the lesion was assessed by *in situ* hybridization for *macrophage scavenger receptor type B* (*Msr-B*). Levels of *Msr-B*-expressing cells did not differ between the groups at 7 and 14 dpl, indicating that rolipram did not affect the macrophage response (Fig 9A–C). Similarly, assessment of Iba1 immunoreactivity did not show any differences in the levels of macrophages within remyelinating lesions (Fig 9D–F).

A–C. Scatter plot showing that the number of *Macrophage scavenger receptor B* (*Msr-B*)-positive macrophages/microglia was comparable between rolipram-treated and control animals. Control *n* = 9, rolipram *n* = 9; ANOVA: *p* > 0.1; control = 146.8 ± 27.97, rolipram = 124.5 ± 18.23. Representative images of sections stained by *in situ* hybridization for *Msr-B* 14 days post lesion induction.
D–F. Similarly, Scatter plot showing that the number of Iba1-positive cells does not differ between the groups 14 days post lesioning. Control *n* = 4; rolipram *n* = 4; *t*-test; *p* < 0.05; control 124.8 ± 24.50 cells/mm^2^, rolipram 104.8 ± 9.022 cells/mm^2^. Representative immunohistochemical Iba1 staining.
G–I. Scatter plot and representative images showing that the phagocytic activity in remyelinating lesions at 7 and 14 dpl is comparable. Phagocytic activity visualized by accumulation of intracellular neutral stained by Oil red O. 7d control *n* = 8, 7 days rolipram *n* = 5, 14 days control *n* = 8, 14 days rolipram *n* = 5; Kruskal–Wallis test: *p* > 0.05. Error bars: SEM. Scale bar = 200 µm, in insets = 50 µm.

To assess whether rolipram treatment induced the phagocytic clearance of myelin breakdown products that accumulate following EB-induced demyelination and inhibit OPC differentiation (Kotter et al, 2006), sections were stained with Oil red O, which revealed neutral lipids accumulating following myelin phagocytosis in macrophages. Blinded ranking analysis did not reveal significant differences between rolipram-treated and control animals (Fig 9G–I).

### Inhibition of Pde4 does not influence injury levels of axons

**Figure 10:**
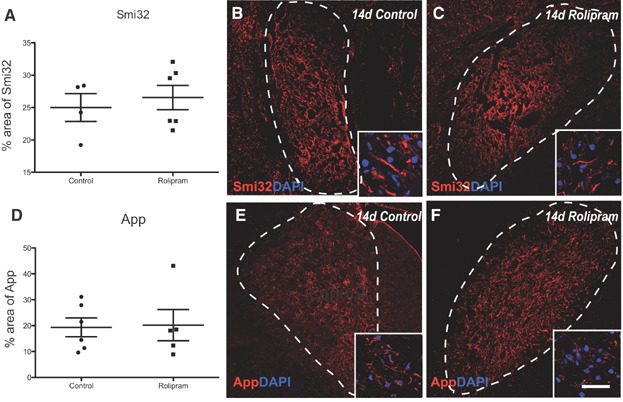
Axonal injury following experimental demyelination is comparable between the groups.

Pde4 is known to modulate axon outgrowth (Hannila & Filbin, 2008) and potentially their survival (Beaumont et al, 2009). We therefore investigated whether rolipram treatment altered the level of axonal damage following stereotactic administration of EB in which some axonal damage occurs (Woodruff & Franklin, 1999), by immunohistochemical analysis of non-phosphorylated neurofilament (Smi32) (Fig 10A–C) and anti-amyloid precursor protein (App) expression (Fig 10D–F). Inhibition of Pde4 did not result in detectable changes in the expression of either marker of axonal injury.

A–C. Scatter blot showing comparable levels of non-phosphorylated neurofilament H (Smi32) in rolipram treated and control animals. Control *n* = 4, rolipram *n* = 6, *t*-test *p* > 0.05; control: 25.00 ± 2.147, rolipram: 26.54 ± 1.874. Representative images of Smi32 immunohistochemistry 14 days post lesion induction.
D–F. Scatter blot demonstrating similar levels of anti-amyloid precursor protein (App) between groups. Control: *n* = 6; 14 days rolipram: *n* = 5, *t*-test *p* > 0.05; control: 19.33 ± 3.647, rolipram: 20.19 ± 6.008. Error bars: SEM. Scale bar = 200 µm, in insets = 50 µm.

## DISCUSSION

The present study identifies cAMP-Erk1/2/p38Mapk-Creb1 signalling as a functionally important intracellular signalling cascade for CNS remyelination that is regulated at the earliest stages of OPC differentiation. These data complement previous studies demonstrating a role for Erk2 and p38Mapk (Chew et al, 2010) as positive regulators of oligodendrocyte differentiation *in vitro* and during developmental myelination. Loss of ERK2 attenuates OPC differentiation and leads to a delay but not a complete arrest in the appearance of differentiated oligodendrocytes *in vivo* (Fyffe-Maricich et al, 2011). Similarly, p38Mapk inhibition decreases OPC differentiation and Mbp expression without effecting either proliferation or survival (Chew et al, 2010). Reporter assay studies have demonstrated that p38MAPK activity up-regulates the activity and/or expression of transcription factors that can bind the 2 kb mouse MBP promoter. However, p38MAPK can also antagonize ERK, JNK, c-Jun phosphorylation. We found that treatment of OPCs in the absence of MAIs with rolipram or cAMP was not able to promote differentiation. It is therefore unclear whether the present strategy would be able to promote developmental myelination.

The finding that OPC differentiation inhibitors present in myelin, which may also form part of the environment in acute demyelinating lesions, negatively regulate cAMP-p38Mapk-Creb1 signalling supports the notion that Mapk signalling plays a functional role in the process of CNS remyelination. More importantly, we found that increasing intracellular cAMP can overcome the inhibitory effects of MAI and stimulate oligodendrocyte lineage progression.

The sequential nature of the Erk1/2-p38Mapk-Creb1 cascade provides multiple points at which the response are regulated by phosphorylation and dephosphorylation and the possibility of tweaking the pathway by pharmacological intervention as to promote oligodendrocyte differentiation in presence of extracellular inhibitors.

cAMP signalling also plays a major role in the regulation of various other cellular processes. For example, inhibition of Pde4 has been successfully used to stimulate axon regeneration *in vitro* and *in vivo* (Pearse et al, 2004). Pde4 is a cAMP-specific Pde and predominant isoenzyme in the majority of inflammatory cells, with the exception of platelets. Pde4 inhibitors such as rolipram and ibudilast can have immunosuppressive and anti-inflammatory effects (Souness et al, 2000) and can delay the entry of inflammatory cells into the CNS in experimental autoimmune disease (EAE) (Fujimoto et al, 1999; Jung et al, 1996; Martinez et al, 1999). However, it is unlikely that these properties are relevant for the present study because the dose of rolipram that was used to enhance remyelination in the present study (0.5 mg/kg/day) is much lower than the dose at which rolipram is effective as an anti-inflammatory agent (3 mg/kg/day) up to 10 mg/kg) (Buttini et al, 1997; Francischi et al, 2000; Laemont et al, 1999). This view is further supported by the absence of changes in the number of cells of the innate immune system and the comparable levels of cell proliferation, phagocytic activity, secretion of Il1-β and numbers of lymphocytes. Furthermore, the levels of axonal damage were not altered. Additionally, we demonstrate that newly formed myelin sheaths were thicker in rolipram treated group compared to control group. However, Pde4 inhibition did not have any effect on spared myelin.

Rolipram treatment did not alter the number of Pdgfr-α positive, immature OPCs in remyelinating lesions. This is what one would predict for a treatment that specifically targets OPC differentiation since the number of immature OPCs in the lesion and the number of mature oligodendrocytes is regulated independently (*e.g*. Arnett et al, 2004). Nevertheless, the number of primed Nkx2.2-positive OPCs at day 7 increased, indicating a response of OPCs to rolipram treatment. As in previous studies, the increase in Nkx2.2 expression was transient and rapidly downregulated when OPCs differentiated into fully mature oligodendrocytes (Fancy et al, 2004). More importantly, rolipram treatment increased the number of Plp-positive cells indicative of mature oligodendrocytes 14 days post lesions induction as compared to controls. Since in experimental model used in this study all lesions will eventually remyelinate, these finding indicate that rolipram accelerates priming and differentiation of OPCs into mature oligodendrocytes.

The potential for clinical translation of the present findings is emphasized by a recent randomized controlled trial that investigated the effects of the Pde4 inhibitor ibudilast on relapse-remitting MS (Barkhof et al, 2010). A number of studies that have demonstrated beneficial effects of Pde4 inhibitors on the development of EAE; this particular study (Barkhof et al, 2010) was designed to assess the disease-modulating properties of ibudilast as an alternative to current immunomodulatory treatments based on interferon-β. The study indicated treatment with a Pde4 inhibitor was safe and well tolerated. The primary endpoint of the study was the cumulative number of newly active lesions on bimonthly brain MRI over 12 months as a measure of disease activity. However, similar to the present findings, inhibition of Pde4 did not affect the inflammation-mediated formation of new active lesions and the rate of relapses remained unchanged. However, the authors reported significant beneficial effects of ibudilast with respect to percent brain volume change, and over a 2-year treatment period there were fewer patients with confirmed progression on the Expanded Disability Status Scale (EDSS) (Barkhof et al, 2010). These beneficial effects of ibudilast were attributed to unknown ‘neuroprotective’ effects resulting from inhibition of Pde4 (Barkhof et al, 2010). Based on the present results it could be hypothesized that this effect may in part be attributable to enhanced remyelination. The ibudilast trial was not designed to study remyelination in MS patients. However, the authors reported a significant reduction in the proportion of active lesions that evolved into persistent ‘black holes’ on T1-weighted MRI images. Although the pathological correlate of black holes is not entirely clear (van Waesberghe et al, 1999), a recent study characterizing T1 black holes in demyelinating Theiler's Murine Encephalitis Virus infection (Pirko et al, 2004) indicated that the black holes might resolve as result of a regenerative response. Thus, one might speculate that at least part of the observations recorded in the ibudilast trial may have been due to enhanced remyelination.

In conclusion, our results suggest that pharmacological inhibition of Pde4 represents a potent and clinically accessible strategy to promote the endogenous efforts of myelin regeneration.

## MATERIALS AND METHODS

### Preparation of primary OPC cultures

Primary OPC cultures were isolated from neonatal Sprague Dawley (postnatal day 0–2) rat forebrains following a standard protocol (Baer et al, 2009). Differentiation was induced by Sato's medium supplemented with 0.5% foetal calf serum (FCS). For all *in vitro* experiments only cultures with ≥94% purity were used.

### Preparation of myelin membrane substrates and myelin protein extracts

Myelin was purified following two rounds of discontinuous density gradient centrifugation and osmotic disintegration as described previously (Baer et al, 2009; Norton & Poduslo, 1973). The MPE was prepared by resuspending the myelin pellets in 1% *N*-octyl β-D-glucopyranoside, 0.2 M sodium phosphate pH 6.8, 0.1 M Na_2_SO_4_ and 1 mM EDTA, and incubated at 23°C for 2 h. Following an ultracentrifugation step (1,00,000 × *g*, 18°C, 30 min) the supernatants were collected and stored at −80°C until further usage (Syed et al, 2008). To test OPC differentiation, slides were coated with MPE following application of PLL.

### Increasing intracellular levels of cAMP

To increase cAMP levels during OPC differentiation in the presence of MAI the OPCs plated on MPE and control (PLL) substrate were treated with the membrane-permeable cAMP analogue dbcAMP (Sigma–Aldrich). Alternatively, hydrolysis of cAMP was prevented by inhibition of Pde4 using selective small molecule inhibitors. Drugs tested included rolipram (Sigma–Aldrich), milrinone (Sigma–Aldrich), irsogladine (Santa Cruz), zaprinast (Sigma–Aldrich) and rottlerin (Millipore), which were dissolved according to the manufacturers' instructions and added to Sato's medium. OPCs were differentiated for 48 h and then fixed in 4% paraformaldehyde (PFA) for immunocytochemistry. A minimum of three independent experiments was conducted.

### Pharmacological inhibition of Erk1/2, p38Mapk and Creb1

The following pharmacological inhibitors were used in the study at the final concentrations indicated: Erk1/2 inhibitor (U0126, 5 µM, Cell Signaling), p38Mapk inhibitor (SB 203580, 10 µM, Calbiochem), CBP-CREB interaction inhibitor (1.5 µM, Calbiochem).

### Immunocytochemical analysis *in vitro*

OPCs were seeded onto PLL- or MPE-coated eight-well chamber slides (2 × 10^4^ cells/well). Following 2 days of differentiation, cells were stained with anti-O4 (1:100; Millipore Corporation), anti-Mbp (1:300, Millipore Corporation) and anti-A2B5 (1:100; Millipore Corporation) antibodies (Alexa 488/Alexa555-conjugated secondary antibody 1:300 dilution, Invitrogen) (Baer et al, 2009; Syed et al, 2011). To assess OPC differentiation, the percentage of O4/Mbp-positive cells relative to >100 DAPI-stained nuclei per experiment for each condition and each experiment in randomly selected eye fields was determined, with the investigator blind to treatment group. To assess the morphological phenotype of OPCs, >100 A2B5-stained cells were categorized as follows: stage I: mono/bipolar; stage II: multipolar, primary branched; stage III: multi-polar, secondary branched; stage IV: secondary branched cells with membranous processes. Following immunocytochemistry, cells were mounted with Prolong gold antifade mounting medium (Invitrogen). Cells were visualized and digitalized at ambient temperature on a LSM 700 confocal microscope (Zeiss) at 20× and 40× magnification using Zen Application software (Zeiss).

### *In situ* proximity ligation assay

Two days after induction of differentiation, oligodendrocytes were fixed and incubated with primary antibodies raised in rabbit against p-Erk1/2 or p-p38Mapk and antibodies raised in mouse against p-Creb1 according to the manufacturer's protocol (1:150). After three washes in phosphate-buffered saline (PBS), the samples were incubated with PLA probes (species-specific secondary antibodies conjugated with a unique short DNA strand). Close proximity of PLA probes (<40 nm) results in direct interaction by subsequent addition of further DNA oligonucleotides by enzymatic ligation. Subsequent rolling circle amplification using a polymerase results in several hundredfold replication of DNA circles. Fluorescently labelled complementary oligonucleotide probes are then used to visualize the nucleotide polymers. Single molecule amplification products can be visualized as distinct bright signal using a confocal microscope. Negative controls included incubation of cells with antibodies against each of the proteins assessed in the absence of antibodies against the proposed binding partner. Slides were mounted on mounting medium containing 4′,6-diamidino-2-phenylindole (DAPI, Olink Biosciences) and assessed on a confocal microscope. PLA signals are quantifiable and this has been conducted by counting the number stained ‘particles’ in ImageJ.

### Assessment of cell viability and apoptosis

Fragmented DNA was detected by TUNEL assay (Promega) and the percentage of apoptotic nuclei was determined (Baer et al, 2009).

### Microarray analysis

RNA was isolated from the oligodendrocytes immediately after shake off and from those which were differentiated for 4 and 12 h in Sato's medium containing 0.5% FCS using an RNeasy Mini Kit (Qiagen). The quality of the RNA was checked with RNA Nano Chips (Agilent) on an Agilent 2100 Bioanalyzer (Agilent). Additionally, the RNA was purified using an RNeasy kit (Qiagen). The RNA was then prepared and hybridized to the Rat Exon 1.0ST array (Affymetrix) following protocols provided by the manufacturer. All raw microarray data are available in MIAME format from the Geo database (Accession: GSE50042 ID: 200050042).

Raw Affymetrix: CEL files were background-corrected, normalized and summarized using the RMA algorithm (Irizarry et al, 2003) as implemented in the Affymetrix PowerTools software (http://www.affymetrix.com/partners_programs/programs/developer/tools/powertools.affx). Probe-sets that were not called present (Affymetrix signal detection statistic, *p* < 0.001) in all profiles of at least one sample group were excluded from analysis. Processed data was imported into the R statistical programming environment (http://www.r-project.org) and differential expression between sample groups assessed using the moderated *t*-test (Smyth, 2004) as implemented in the limma package for the Bioconductor suite of R bioinformatics software (http://bioconductor.org). In order to correct for hypothesis testing on such a scale, *p*-values relating to differential expression were corrected for multiple testing using FDR correction (Storey & Tibshirani, 2003). Differential expression across sample groups was deemed significant at an FDR of 5% (*q* < 0.05).

### Quantitative reverse transcriptase PCR

Total RNA was extracted using an RNeasy Mini Kit (Qiagen). qRT-PCR was conducted as previously outlined on (Baer et al, 2009) an Applied Biosystems 7500HT Fast Real-time PCR system. Values are represented as Mbp/β2-microglobulin ratios. Triplicate measurements were made on three biological replicates.

### Immunoprecipitation and Western blotting

Cells were chilled on ice, washed twice with ice-cold PBS and lysed in ice-cold immunoprecipitation (IP) buffer supplemented with protein and protease inhibitors (Thermoscientific). Soluble lysates were prepared by centrifugation at 14,000 × *g* for 20 min at 4°C, and then incubated with anti-Erk1/2, anti-p38Mapk or anti-Creb antibodies (Cell Signaling) for 2 h on an orbital shaker. Protein A/G agarose beads (Santa Cruz) were added and lysate was placed on rocking platform overnight at 4°C. Immunocomplexes were collected by centrifugation and washed several times in ice-cold IP buffer. Immunoprecipitated proteins were resolved by 12% SDS–PAGE, and immunoblot analysis was performed according to the manufacturer's instructions (Invitrogen). The blots were incubated overnight with antibodies against p-Erk1/2, p-p38Mapk or p-Creb. Immunocomplexes were detected by enhanced chemiluminescence. Following this, the blots blot were stripped, blocked and reprobed with anti-Erk1/2, anti-p38Mapk or anti-Creb (Cell Signaling) antibodies.

### Induction of focal demyelination

All experiments were conducted in accordance with animal welfare regulations of the UK Home Office and institutional guidelines for animal care and handling (Project license number: 80/228). Female Sprague Dawley rats (180–200 g) were anaesthetized using 4% isoflurane in oxygen and positioned in a stereotactic instrument. Demyelination was induced bilaterally by stereotactic injection of EB (0.01%, 4 µl) into the CCP (10.4 mm caudal, ± 2.6 mm lateral, and 7.07 mm ventral to the bregma (Woodruff & Franklin, 1999). Rolipram (Sigma–Aldrich) in saline:DMSO 50:50 was administered at a concentration of 0.5 mg/kg/day, and PBS as a control, by implantation of subcutaneous osmotic minipumps (2ML2, ALZET) 3 days after surgical lesion induction. Animals were sacrificed after 7 and 14 dpl. To rule out effects attributable to the size of the lesion, the density of Plp-positive cells was assessed in PBS-treated animals of this and previous studies (Syed et al, 2011) by linear regression (GraphPad, Prism). The inclusion of lesions <0.4 mm^2^, which were strongly populated with Plp-positive cells, results in a significant non-zero slope indicating an effect of the lesion size on the cell density. This may be explained by the fact that in small lesions recruitment of OPCs and the clearance of myelin debris is more efficient and thus remyelination becomes more efficient compared to larger lesions. No significant correlation between the size of the lesion and the density of Plp-positive cells was observed in lesions >0.4 mm^2^ area. Consequently, only lesions >0.4 mm^2^ were included in the analysis.

### *In situ* hybridization

The expression of a number of marker mRNA species in demyelinated lesions was examined by *in situ* hybridization with digoxigenin-labelled cRNA probes. Animals were perfused with 4% PFA via the left ventricle. The tissue was extracted, post-fixed in 4% PFA, cryoprotected in 30% sucrose and snap frozen. *In situ* hybridizations were conducted on cryostat sections (15 µm) using established protocols (Kotter et al, 2006).

Lesions were identified on digital images of solochrome cyanine-stained sections, and the lesion area was determined using ImageJ 1.43b. The same program was used to determine the number of stained cells within the lesions on digitized adjacent sections. All analyses were conducted investigator blind to treatment group.

### Immunohistochemical analysis *in vivo*

Immunohistochemistry were performed on 10 µm-thick sections of 4% PFA-fixed tissue. Antigen retrieval was performed with 0.01 M citrate buffer (Dako) at pH 6.0 for 20 min in a 95°C water bath. Slides were allowed to cool for another 20 min, followed by rinsing in PBS. Sections were then incubated with 10% inactivated normal donkey serum (Abcam) for 60 min at room temperature to block background staining. The cell membrane was permeabilized for 15 min in 0.1% Triton X-100 in serum. Staining with the following antibodies was performed: p-Erk1/2 (1:300), p-p38Mapk (1:300), p-Creb (1:300, Cell Signaling), Nkx2.2 (1:300, U. Iowa), Olig2 (1:1000), App(1:1000, Millipore), Iba1 (1:500), SMI32 (1:2000, Convance) and Ki67 (1:500, Dako). Sections were incubated with primary antibody overnight, washed three times in PBS and then incubated with appropriate Alexa 488- or 594-conjugated secondary antibodies (Invitrogen) for 2 h at room temperature. They were then counterstained with DAPI (Sigma–Aldrich). Prolong gold antifade mounting medium (Invitrogen) was used to mount the slides. Negative controls were performed by replacing the primary antibody with serum or by incubating sections with each primary antibody followed by the corresponding secondary antibody that is not specific to the species it was raised against. Slides were examined in a fluorescence microscope (Olympus), and images were taken by confocal microscopy (Zeiss). All quantifications were conducted with the investigators blinded to the treatment group. Iba1-positive microglia/macrophages were manually counted on digitised sections. Automated counting was conducted for Ki67/Olig2 stained cells. To quantify APP and SMI32 positive axons in the lesions the area comprised by immunostaining above a calculated threshold following binary conversion relative to the entire lesion area was calculated. Only lesions >0.4 mm^2^ were included in the analysis. Data are given as mean ± SEM and statistically analysed. Two sections per marker per animal were analysed and minimum of four animals were used.

### Oil Red O staining

Oil Red O (Sigma–Aldrich) working solution was prepared by adding 20 ml dH_2_O to 30 ml 1% Oil Red O in isopropanol. Random sections from groups were stained for 10 min then washed for 4 min and counterstained in Carrazi's haematoxylin for 4 min. Following a 4 min wash in H_2_O, the sections were differentiated in 0.5% aqueous hydrochloric acid for 7 s and again washed in water for 10 min. Finally, the slides were mounted using an aqueous mounting medium. Representative images of Oil Red O stained lesions were digitized and blindly ranked with the highest staining density receiving the highest rank value. Analyses were conducted with investigator blinded to treatment group.

### Histological analysis of remyelination

To assess the extent of remyelination the tissue, was fixed in 4% glutaraldehyde, osmicated and processed into resin (TAAB Laboratories) (Woodruff & Franklin, 1999). Sections (1 µm) were stained with 1% toluidine blue. The extent of remyelination was then assessed by light and electron (see below) microscopy. Based on the thickness (or absence) of myelin sheaths demyelinated axons, axons bearing native myelin sheaths, and remyelinated axons on light and electron micrographs can unambiguously be distinguished (Blakemore, 1974). Lesions were ranked according to the extent of remyelination by two blinded investigators and statistically analysed using a two-tailed Mann–Whitney test.

### Electron microscopy

Ultra-thin sections (50 nm) containing the lesions were stained with aqueous 4% uranylacetate and lead citrate. The sections were visualized on an electron microscope (Hitachi H-600 Electron Microscope). Demyelinated versus remyelinated axons were manually counted on a minimum of 10 micrographs randomly taken from the lesion border. Ratios of remyelinated and demyelinated versus the total number of axons were analysed using Student *t*-test. *G*-ratios (=the ratio of axon circumference to myelin circumference) of oligodendrocyte-remyelinated axons were calculated using ImageJ and statistically analysed. (Lower *G*-ratio values indicate thicker myelin sheaths).

### Statistical analysis

Data were analysed using GraphPad software (Prism). Multiple group comparisons were conducted using one-way ANOVA followed by Dunnett's *post test*. Analysis of qRT-PCR data was done using two-tailed Student's *t*-tests. For rank analysis a two-tailed nonparametric Mann–Whitney *U* test was used.

### The paper explained

**PROBLEM:**

MS is a devastating disease that involves the loss of myelin, the protective sheaths around axons that enhance impulse conduction. The increasing effectiveness of new drugs that slow the progression of MS has opened up opportunities for medicines that enhance remyelination and potentially slow disease progression even further. Although several new targets for therapeutic enhancement of remyelination have emerged, few lend themselves readily to conventional drug development.

**RESULTS:**

Here, we used transcription-profiling approaches to identify mitogen-activated protein kinase (Mapk) signalling as an important regulator involved in the differentiation of OPCs into oligodendrocytes, the cells that form myelin. We show that activation of Mapk signalling by elevation of intracellular levels of the intracellular second messenger cAMP using either a cAMP analogue or inhibitors of the cAMP-hydrolysing enzyme phosphodiesterase-4 (Pde4) enhances OPC differentiation in tissue culture. Finally, we demonstrate that systemic delivery of a Pde4 inhibitor leads to enhanced differentiation of OPCs within focal areas of toxin-induced demyelination and thus to an acceleration of remyelination.

**IMPACT:**

This study identifies cAMP-Erk1/2/p38Mapk-Creb1 signalling as a functionally important intracellular signalling cascade for CNS remyelination that is regulated at the earliest stages of OPC differentiation. Our results suggest that inhibiting Pde4 could be a way to enhance remyelination in chronic demyelinating diseases, such as MS. Pde4 inhibitors are already approved and used to treat other diseases, so there is great potential for rapid translation of this discovery into clinical practice.

## Author contributions

YAS, AB and MRK designed the experiments; YAS, AB, MPH, GAG, JR and MWBT conducted the experiments; JKH, CZ, MJR and GL contributed new reagents/analytic tools; YAS, AB, MWBT, SM, RJMF and MRK analysed data; and YAS, RJMF and MRK wrote the paper.

## Notes

As a service to our authors and readers, this journal provides supporting information supplied by the authors. Such materials are peer reviewed and may be re-organized for online delivery, but are not copy-edited or typeset. Technical support issues arising from supporting information (other than missing files) should be addressed to the authors.
