# Supplementary material for: Inhibition of phosphodiesterase-4 promotes oligodendrocyte precursor cell differentiation and enhances CNS remyelination
Source: EMBO Mol Med. 2013 Oct 21;5(12):1918–34. doi: 10.1002/emmm.201303123 (PMC3914530; doi:10.1002/emmm.201303123)
Supplement: Supplementary file 1 [file emmm0005-1918-sd1.pdf]

EMBO Molecular Medicine

Inhibition of phosphodiesterase-4 promotes oligodendrocyte precursor cell differentiation and enhances CNS remyelination

Yasir A. Syed, Alexandra Baer, Matthias P. Hofer, Ginez A. González, Jon Rundle, Szymon Myrta, Jeffrey K. Huang, Chao Zhao, Moritz J. Rossner, Matthew W.B. Trotter, Gert Lubec, Robin J.M. Franklin, Mark R. Kotter

Corresponding author: Mark Kotter, University of Cambridge and Robin J.M. Franklin, Wellcome Trust and MRC Cambridge Stem Cell Institute

Review timeline:

Submission date:	03 June 2013
Editorial Decision:	27 June 2013
Revision received:	01 August 2013
Editorial Decision:	09 September 2013
Revision received:	18 September 2013
Accepted:	18 September 2013

Transaction Report:

(Note: With the exception of the correction of typographical or spelling errors that could be a source of ambiguity, letters and reports are not edited. The original formatting of letters and referee reports may not be reflected in this compilation.)

Editor: Céline Carret

1st Editorial Decision 27 June 2013

Thank you for the submission of your manuscript to EMBO Molecular Medicine. While reviewers 1 and 3 delivered their evaluations in a timely manner, we did not receive as yet the last reviewers' input. As the evaluations from the first two reviewers are consistent, and a further delay cannot be justified, I have decided to proceed based on these evaluations.

If in the meanwhile we should receive the other review and only if it raises significant caveats, these will need to be taken into consideration. We would not, however, ask you to comply with any further-reaching requests.

You will see that while both reviewers are generally supportive of your work and underline its potential interest, they also raise a number of specific concerns that prevent us from considering publication at this time. However, we feel that the required revisions are addressable and would significantly improve the study. Referees 1 and 2 request additional details and explanations, particularly to improve the in vivo significance of the findings (ref.1). Referee 2 also suggests adding a few experiments and better controls to strengthen the data and better support the claims.

Should you be able to address the raised concerns with additional experiments where appropriate, we would be willing to consider a revised manuscript.

Please note that it is EMBO Molecular Medicine policy to allow a single round of revision in order to avoid the delayed publication of research findings. Consequently, acceptance or rejection of the manuscript will depend on the completeness of your responses included in the next version of the manuscript.

EMBO Molecular Medicine has a "scooping protection" policy, whereby similar findings that are published by others during review or revision are not a criterion for rejection. Should you decide to submit a revised version, I do ask that you get in touch after three months if you have not completed it, to update us on the status.

Please also contact us as soon as possible if similar work is published elsewhere. If other work is published we may not be able to extend the revision period beyond three months.

I look forward to receiving your revised manuscript.

***** Reviewer's comments *****

Referee #1 (Remarks):

The authors performed a careful study on the effects of phosphodiesterase inhibition on OPC differentiation and remyelination. The study nicely combines different in vitro and in vivo approaches. Howevre, there are several issues that raise questions and criticism that should be addressed by the authors:
1. The authors several times mention in the manuscript that the presence of myelin inhibitory factors is likely to impair remyelination in early stage MS lesions. This is however not the case. In early stage MS lesions, remyelination works, it is impaired significantly in chronic disease stages.
2. The in vitro data are convincing and nicely demonsteate the involvement of the mapk pathway in OPC differentiation. However, these in vitro findings only marginally translate into an improved remyelination in vitro. Although there is at least some remyelination in control animals, why is there no change in NKx2.2 and PLP mRNA expressing cells. In rolipram treated animals. is the increase in NKx2.2 positive only temporary and does this explain the limited improvement in remyelination? It would be useful to see the numbers of Olig2+Ki-67+ positive cells also at day 7.
3. The data for remyelination in vivo are not convincing. The blind rank analysis is not explained very well in the Methods section and appears to be too subjective. The changes in the number of demyelinated and remyelinated axons is significant but at a very low scale. The question for me is whether the mapk pathway would really be such a good target for remyelination in vivo.
4. The g-ratio data are hard to interpret. g-ratio is higher in controls than in rolipram treated animals. Does that mean that the demyelinated axons were included in the measurements in the controls or is the myelin really thicker in rolipram treated animals. This is not clear from the methods section.
5. Innate immune response: the authors should give numners of IBA-1 positive cells and not fluorescence intensities. Oil red O is a very late marker of myelin phagocytosis. Since the authors looked at earlier stages, they should be able to see myelin protein positive macrophages. Again here showing rank values is not appropiate, numbers of cells per mm2 is the appropiate method.
6. The same concerns APP and SMI32 positive axons. the authors should numbers of positively stained axons not intensities.
7. In the discussion, the ibudilast trial is a bit overinterpreted in the light of the data presented here.

Referee #3 (Remarks):

"Inhibition of phoshodiesterase-4 promotes oligodendrocyte precursor cell differentiation and enhances CNS myelination" Syed and Baer et al.
It is well established that certain aspects of MAPK signaling play a significant role in oligodendrocyte biology by regulating its proliferation and myelin production. One mechanism regulating the differentiation of immature to mature OPCs is controlled by p38 MAPK (Chew et al., J. Neurosci 2010) whereas the proliferation of OPCs and the amount of actual myelin protein

production by oligodendrocytes is under the control of ERK1/2 MAPK (Ishii et al., J. Neurosci 2013). The authors here are investigating the early signaling events associated with the OPC proliferation and differentiation. They used transcription-profiling to identify members of the MAPK family that are being differentially regulated. They compared their gene expression profile data with previously published data of CNS remyelination and concluded that MAPK signaling could be a role in CNS remyelination. They hypothesized that modulating levels of cAMP by inhibiting Pde-4 would alleviate cell signaling associated with the presence of myelin-associated inhibitors (MAI). The results support that inhibition of Pde-4 positively regulates OPC differentiation via crosstalk with the MAPK-CREB1 pathway in their model of experimental demyelination.
Major strengths of this manuscript are:
1. The authors delineate the role and mechanisms of complex upstream signaling pathways controlling the activation of MAPK during OPC differentiation
2. They further go on to show the importance of the in-vivo Pde-4 signaling cascade during OPC differentiation and in experimental demyelination.

The following are recommendations that could further strengthen the manuscript:

Comments regarding the figures:
1. The western blots shown in Fig.1A depict results following MPE treatment and are shown in support of a block in phosphorylation of ERK1/2 but it also appears that the total levels of ERK1/2 are also decreasing. The authors either need to address the reason for this decrease in the text or provide a better blot; Does MPE treatment destabilize the ERK protein levels? Also in Fig1.C. Decrease in pCREB1 activation by MPE is small; a blot showing a stronger decrease would be more convincing.
2. Fig. 3. Depicting interaction of phospho-ERK1/2 and phospho-p38 with CREB1 via the "in situ protein interaction assay" is qualitative at best. The authors could provide quantification of the fluorescent signals reflecting the extent/degree of interaction to strengthen their claims of a direct interaction between these proteins in the OPCs. An alternative experiment could be performed in the event the quantification of signals proves difficult; the authors could perform co-IPs using tagged CREB1 constructs transfected into OPCs to immunoprecipitate the interacting ERK1/2 and p38 kinases under differentiating conditions to show protein-protein interaction.
3. Fig. 4 is missing essential controls. Authors show that the negative aspects of MPE signaling on OPCs can be rescued by treating the cells with db-cAMP and Rolipram. The authors should provide data to show that treatment of OPCs with db-cAMP alone and Rolipram alone does/does not influence the maturation of O4 positive and MBP positive cells to rule out non-specific effects of the drug.
4. Fig. 5A: the blot showing the activation of ERK1/2 following Pde-4 inhibition would be more convincing with a better blot. Also, the decrease in total levels of ERK1/2 is again observed following MPE treatment; the authors need to address this (see comment 1 above).
Minor Comments to strengthen the Discussion:
1. Did the authors attempt a transcription profile analysis of OPCs following their culture with MAIs to assess whether this treatment leads to down regulation of MAPK signaling components? It would be interesting to determine whether up-regulation of Pde-4 is observed following MPE treatment in OPCs and this would further strengthen the results of the manuscript.
2. In the figure legend for Fig.1 authors put forward a model where an increase in cAMP activity leads to PKA, p38MAPK and CREB1 activation; both p38 and CREB1 activation are assayed but PKA activation is not assessed. Why?
3. It is not clear from the text as to what the authors are referring to the difference between OPC "activation" Vs "differentiation" (See Paragraph 1 on Pg.8). Does OPC activation mean the "priming" of OPC for differentiation? Perhaps using better terminology would make it less confusing.
4. The manuscript presents data, which suggests that Pde-4 inhibition promotes remyelination yet there is no mention anywhere in the manuscript about the role of Pde-4 in the normal development of OPCs, if it plays any. The authors should address this point either in the results or the discussion section.
5. The downstream signaling events associated with the activation of p38MAPK and ERK1/2 MAPK have parallels and as well as similarities. Clearly, the literature suggests that p38MAPK and ERK1/2 MAPK fulfill different roles in oligodendrocyte biology. The results presented in the manuscript also suggest this; the authors observe a stronger differentiation block on OPCs when

p38MAPK is inhibited Vs ERK1/2 (Fig2A-C Vs Fig2D-F). The authors do not highlight this difference between the two signaling events and what effects it may have on oligodendrocytes anywhere in the manuscript; they should add a comment either in the discussion or results section to address this.
Specific Comments:
1. Last paragraph of Pg.5 discusses the results of the "protein ligation assay" as shown in Fig.3. But the assay is referred to as "proximity ligation assay (PLA)" in the figure legend; this is confusing, please use the same terminology.
2. Pg.8 third paragraph line1 please define "CCP".
3. Pg.9 First paragraph, figure 9A-C is mislabeled as Fig. 6A-C.
4. Pg.10 Third paragraph "tweaking" is misspelled as "tweeking".

1st Revision - authors' response 01 August 2013

Reviewer 1

1. *The authors several times mention in the manuscript that the presence of myelin inhibitory factors is likely to impair remyelination in early stage MS lesions. This is however not the case. In early stage MS lesions, remyelination works, it is impaired significantly in chronic disease stages.*

We are grateful to the reviewer for giving us the opportunity to clarify this important point. All lesions, whatever their fate, must start as acute lesions. Some will go on to remyelinate and some will remain demyelinated (and some mixed). Thus, while it is true that the initiation of remyelination, when successful, is a feature of an acute lesion, it also follows that a lesion becomes a chronic demyelinated lesion because something has gone wrong in the acute phase (such as failure to adequately clear myelin debris). In other words, one cannot think of acute and chronic lesions as separate entities but as chronologically linked – the features of the chronic phase arising because of events occurring in the acute phase. We have revised the manuscript bearing in mind this distinction and specifically refer to “early stage MS lesions” and not to “lesions at early stages of MS”.

2. *The in vitro data are convincing and nicely demonstrate the involvement of the mapk pathway in OPC differentiation. However, these in vitro findings only marginally translate into an improved remyelination in vitro (presumably the reviewer means in vivo here - authors). Although there is at least some remyelination in control animals, why is there no change in NKx2.2 and PLP mRNA expressing cells. In rolipram treated animals. is the increase in NKx2.2 positive only temporary and does this explain the limited improvement in remyelination? It would be useful to see the numbers of Olig2+Ki-67+ positive cells also at day 7.*

The difference in the degree of phenotypic change seen in vitro and in vivo is not surprising to us – it is frequently the case that a stronger effect is seen in the simple reductionist context of the tissue culture dish compared to the complex multi-variant in vivo environment. Nevertheless, it is worth remembering that our in vivo model of demyelination, like many other models, is a model that will eventually undergo full remyelination (albeit in an age-dependent manner) and so what we report is an acceleration of remyelination brought about by the earlier induction of progenitor differentiation.

Nkx2.2 mRNA reaches a transient peak expression at the start of OPCs differentiation in remyelinating lesions. In mature oligodendrocytes Nkx2.2 mRNA expression is decreased (Fancy et al., 2004). Increased expression of Nkx2.2 at early time points of remyelination is therefore consistent with previous observations.

The expression of Plp, a marker of mature oligodendrocytes, in rolipram treated animals follows a pattern that the in vitro findings predict: rolipram treatment induced a significant increase in the number of Plp expressing cells at day 14 as compared to controls indicating that rolipram promoted OPC differentiation.

Instead of Olig2+Ki67+ cells (essentially corresponding to the proliferating fraction of the entire oligodendrocyte lineage labelled with Olig2) we have assessed the number of immature OPCs by ISH for PDGFR-a. This did not reveal any differences between the control and the treatment groups. This is precisely what one would predict for a treatment effect that regulates differentiation since the number of OPCs in the lesion and the number of oligodendrocytes is differently regulated (it is not the case that differentiation of OPC into oligodendorcytes reduces their number since the number of OPC is controlled by the availability of survival factor). We have modified the discussion to address this point.

3. *The data for remyelination in vivo are not convincing. The blind rank analysis is not explained very well in the Methods section and appears to be too subjective. The changes in the number of demyelinated and remyelinated axons is significant but at a very low scale. (...)*

We thank the reviewer for bringing to our attention the description of the methodology used to determine the degree of remyelination. This has been now addressed in a revised account of the methods we used and why.

In the past 15 years we have evaluated various methods of assessing the extent of CNS remyelination in experimental models of demyelination. These include immunohistochemical and quantitative mRNA-based approaches as well as this involving light and electron microscopical of resin embedded tissue. We have compared manual counts of axons on electron micrographs with light microscopical analysis of the same lesions and found that light microscopical quantification does not differ significantly. However, we found that the most reproducible way of assessing the effects of a particular intervention on remyelination is by investigator blinded rank analysis followed by analysis using Mann Whitney U test. Histological assessments are conducted by a minimum of two investigators independently and are highly reproducible. One of the most robust tests for comparison of two groups with limited sample size is a Mann Whitney U test. This test ultimately treats numerical data as rank values - and so in terms of addressing whether an intervention has significantly altered remyelination there are no advantages to use quantitative source data.

With respect to the "low scale" of the differences observed we have emphasized in the revised manuscript that, as described above, the model of remyelination that we have used, in common with nearly all other experimental models, will eventually undergo full remyelination. Therefore, what we are achieving is a change in rate of a dynamic process (which may well be precisely what is required therapeutically) rather than extent of remyelination.

4. *The g-ratio data are hard to interpret. g-ratio is higher in controls than in rolipram treated animals. Does that mean that the demyelinated axons were included in the measurements in the controls or is the myelin really thicker in rolipram treated animals. This is not clear from the methods section.*

We are grateful to the reviewer for pointing out the ambiguities in the way we have presented this. In resin section of toxin-induced spinal cord white matter demyelination the distinction between myelinated and remyelinated axons is unambiguous – the myelinated axons have markedly thicker myelin sheaths (and therefore lower g ratios. Only remyelinated axons were included in the analysis. For increased clarity we have now included base-line G-ratios of native (non-demyelinated) myelin sheaths of both groups on the graphs.

5. *Innate immune response: the authors should give numbers of IBA-1 positive cells and not fluorescence intensities. Oil red O is a very late marker of myelin phagocytosis. Since the authors looked at earlier stages, they should be able to see myelin protein positive macrophages. Again here showing rank values is not appropriate, numbers of cells per mm2 is the appropriate method.*

As suggested by the reviewer, we have now counted the number of IBA-1 positive cells and this data is now presented in figure 9D.

6. *The same concerns APP and SMI32 positive axons. The authors should numbers of positively stained axons not intensities.*

We can see the argument that fluorescence levels, which may not follow a linear distribution, potentially are not the best way of quantifying axonal damage. On the other hand, counting and quantifying stretches of various axons on a section may also have its shortfalls and is technically challenging. Encompassing the reviewer's concerns have therefore revised the strategy and now present the data as the area comprised by immunostaining above a calculated threshold following binary conversion relative to the entire lesion area. This is the closest feasible approximation to the requested "number of positively stained axons".

7. *In the discussion, the ibudilast trial is a bit overinterpreted in the light of the data presented here.*

We agree and have changed the wording to reflect the hypothetical nature of this interpretation.

Reviewer 3

1. *The western blots shown in Fig.1A depict results following MPE treatment and are shown in support of a block in phosphorylation of ERK1/2 but it also appears that the total levels of ERK1/2 are also decreasing. The authors either need to address the reason for this decrease in the text or provide a better blot; Does MPE treatment destabilize the ERK protein levels? Also in Fig1.C. Decrease in pCREB1 activation by MPE is small; a blot showing a stronger decrease would be more convincing.*

Fig. 1A) In response to this comment we have specifically compared the expression of total Erk1/2 across the samples and did not find any differences. We therefore have changed the blot to better reflect the experimental findings.
Fig1.C) We agree with the reviewer in principle. However, we tried to include representative blots rather than 'best' blots to better convey the authentic nature of our data.

2. *Fig. 3. Depicting interaction of phospho-ERK1/2 and phospho-p38 with CREB1 via the "in situ protein interaction assay" is qualitative at best. The authors could provide quantification of the fluorescent signals reflecting the extent/degree of interaction to strengthen their claims of a direct interaction between these proteins in the OPCs. An alternative experiment could be performed in the event the quantification of signals proves difficult; the authors could perform co-IPs using tagged CREB1 constructs transfected into OPCs to immunoprecipitate the interacting ERK1/2 and p38 kinases under differentiating conditions to show protein-protein interaction.*

We thank the reviewer for this comment. We have conducted both immuno-precipitations and Duolink assays in the past and found that the in situ protein ligation assays are not only more reliable but also better illustrate the interaction. Furthermore, proximity ligation assays are quantifiable and we have included this data in the revised manuscript.

3. *Fig. 4 is missing essential controls. Authors show that the negative aspects of MPE signaling on OPCs can be rescued by treating the cells with db-cAMP and Rolipram. The authors should provide data to show that treatment of OPCs with db-cAMP alone and Rolipram alone does/does not influence the maturation of O4 positive and MBP positive cells to rule out non-specific effects of the drug.*

This is a valid point. The rolipram control on PLL has now been included, indicating that rolipram cannot promote differentiation in the absence of inhibitory substrates.

4. *Fig. 5A: the blot showing the activation of ERK1/2 following Pde-4 inhibition would be more convincing with a better blot. Also, the decrease in total levels of ERK1/2 is again observed following MPE treatment; the authors need to address this (see comment 1 above).*

See comments above. A better blot has now been included.

Minor Comments to strengthen the Discussion:

1. *Did the authors attempt a transcription profile analysis of OPCs following their culture with MAIs to assess whether this treatment leads to down regulation of MAPK signaling components? It would be interesting to determine whether up-regulation of Pde-4 is observed following MPE treatment in OPCs and this would further strengthen the results of the manuscript.*

This is a very interesting comment. We have indeed conducted a microarray experiment comparing OPCs plated on PLL control substrates with OPCs plated on myelin substrates. We haven now included a heat map showing changes with respect to Mapk related genes at 4h following induction of differentiation. Furthermore, we show that Pde-4 isoforms do not seem to be regulated by the presence of MAI within the first 4h.

2. *In the figure legend for Fig.1 authors put forward a model where an increase in cAMP activity leads to PKA, p38MAPK and CREB1 activation; both p38 and CREB1 activation are assayed but PKA activation is not assessed. Why?*

This is again a very valid point. We have removed PKA from the model.

3. *It is not clear from the text as to what the authors are referring to the difference between OPC "activation" Vs "differentiation" (See Paragraph 1 on Pg.8). Does OPC activation mean the "priming" of OPC for differentiation? Perhaps using better terminology would make it less confusing.*

We agree that “activation” may be a bit generic and have now used the term “priming” to refer to the state at the onset of OPC differentiation.

4. *The manuscript presents data, which suggests that Pde-4 inhibition promotes remyelination yet there is no mention anywhere in the manuscript about the role of Pde-4 in the normal development of OPCs, if it plays any. The authors should address this point either in the results or the discussion section.*

The role of Pde-4 on developmental myelination has not yet been assessed in detail. The lack of data is now mentioned in the discussion.

5. *The downstream signaling events associated with the activation of p38MAPK and ERK1/2 MAPK have parallels and as well as similarities. Clearly, the literature suggests that p38MAPK and ERK1/2 MAPK fulfill different roles in oligodendrocyte biology. The results presented in the manuscript also suggest this; the authors observe a stronger differentiation block on OPCs when p38MAPK is inhibited Vs ERK1/2 (Fig2A-C Vs Fig2D-F). The authors do not highlight this difference between the two signaling events and what effects it may have on oligodendrocytes anywhere in the manuscript; they should add a comment either in the discussion or results section to address this.*

We have changed the discussion accordingly.

Specific Comments:

1. Last paragraph of Pg.5 discusses the results of the "protein ligation assay" as shown in Fig.3. But the assay is referred to as "proximity ligation assay (PLA)" in the figure legend; this is confusing, please use the same terminology.
2. Pg.8 third paragraph line1 please define "CCP".
3. Pg.9 First paragraph, figure 9A-C is mislabeled as Fig. 6A-C.
4. Pg.10 Third paragraph "tweaking" is misspelled as "tweeking".

We thank the reviewer for the comments and have corrected the mistakes accordingly.

2nd Editorial Decision 09 September 2013

Thank you for the submission of your revised manuscript to EMBO Molecular Medicine. We have now received the enclosed reports from the referees that were asked to re-assess it. As you will see the reviewers are now supportive and I am pleased to inform you that we will be able to accept your manuscript pending the following final amendments:

- Please address the minor issues highlighted by referee 2
- Reduce the number of keywords to 5
- remove all text in red font from the article file
- provide an accession number for the microarray dataset. As in our guidelines, data of gene expression experiments described in submitted manuscripts must be deposited in a MIAME-compliant format with one of the public databases. We would therefore ask you to submit your microarray data to the ArrayExpress database maintained by the European Bioinformatics Institute for example or to the GEO database maintained by NCBI.
- as in our guidelines, you must provide an ethical statements for the use of living animals, including the obtention of samples
-regarding the figures, could you please delineate the western blots by a black line. We now encourage the publication of source data, particularly for electrophoretic gels and blots, with the aim of making primary data more accessible and transparent to the reader. Would you be willing to provide a single PDF file comprising the original, uncropped and unprocessed scans of all or key gels used in the figures? These should be labeled with the appropriate figure/panel number, and should have molecular weight markers; further annotation could be useful but is not essential. This PDF will be published online with the article as a supplementary "Source Data" file. If you have any questions regarding this just contact me.

Please submit your revised manuscript within two weeks. I look forward to seeing a revised form of your manuscript as soon as possible.

***** Reviewer's comments *****

Referee #1 (Comments on Novelty/Model System):

The authors responded adaequately and sufficiently to all criticism that was raised by the reviewers.

Referee #1 (Remarks):

All criticism raised by the reviewers was sufficiently dealt with and the manuscript significantly improved.

Referee #3 (Comments on Novelty/Model System):

This study focuses on strategies to enhance myelination which is a major deficit in several major human neurological disorders: multiple sclerosis, cerebral palsy, vascular dementia. The work thus is timely and has broad appeal to both neuroscientists and clinicians.

Referee #3 (Remarks):

"Inhibition of phoshodiesterase-4 promotes oligodendrocyte precursor cell differentiation and enhances CNS myelination" Syed and Baer et al.
The authors have addressed all of my concerns and I would recommend the manuscript for publication.

Specific comments:

1. Fig 4O; "PLL+db-cAMP" is mislabeled as "MPE+db-cAMP"
2. Pg.17 in Materials and Methods section; Paragraph 2, line 2, "95ºC" is misspelled as "95%"

2nd Revision received and accepted 18 September 2013
