# Supplementary material for: Inhibition of phosphodiesterase-4 promotes oligodendrocyte precursor cell differentiation and enhances CNS remyelination
Source: EMBO Mol Med. 2013 Oct 21;5(12):1918–34. doi: 10.1002/emmm.201303123 (PMC3914530; doi:10.1002/emmm.201303123)
Supplement: Supplementary file 2 [file emmm0005-1918-sd2.pdf]

Supporting Information

Inhibition of phosphodiesterase-4 promotes oligodendrocyte precursor cell differentiation and enhances CNS remyelination

Running title: Inhibition of phosphodiesterase-4 promotes CNS remyelination

Yasir A. Syed*[1,2,5], Alexandra Baer*[2,3], Matthias P. Hofer[1], Ginez A. González[1, 4], Jon Rundle[1,4], Szymon Myrta[1], Jeffrey K. Huang[4], Chao Zhao[4], Moritz J. Rossner[6], Matthew W.B. Trotter[1], Gert Lubec[3], Robin J.M. Franklin[4+], Mark R. Kotter[1,2,5+]

Author's affiliations:

1) Wellcome Trust and MRC Cambridge Stem Cell Institute, and Anne McLaren Laboratory for Regenerative Medicine, University of Cambridge, West Forvie Building, Forvie Site, Robinson Way, Cambridge, CB2 0SZ, UK
2) Department of Neurosurgery, Medical University Vienna, Waehringer Guertel 18-20, 1090 Vienna, Austria
3) Department of Pediatrics, Medical University Vienna, Waehringer Guertel 18-20, 1090 Vienna, Austria
4) Wellcome Trust and MRC Cambridge Stem Cell Institute, and Department of Veterinary Medicine, University of Cambridge, Madingley Road, Cambridge, CB3 0ES, UK
5) Max-Planck Institute for Experimental Medicine, Department of Neurogenetics, 37075 Goettingen, Germany
6) Max-Planck- Institute for Experimental Medicine, Research Group 'Gene Expression and Signalling', Department of Neurogenetics, D-37075 Göttingen, Germany

Table of content

1. Suppl. Fig 1 Heat-map representation of some Mapk genes identified by microarray analysis.
2. Suppl. Fig 2 Creb1 activity increases during oligodendrocyte differentiation.
3. Suppl. Fig 3 Treatment with Pde4 inhibitors promote OPC differentiation in the presence of MAI.
4. Suppl. Fig 4 Quantification of apoptotic cells by TUNEL assay.
5. Suppl. Fig 5 Double immunohistochemistry conducted at the peak of the differentiation phase in remyelinating lesions (14 dpl) confirms expression of activated Erk1/2, p38Mapk, and Creb1 in OPCs.
6. Suppl. Fig 6 Rolipram treatment does not change the size of the lesion.
7. Suppl. Fig. 7 Experimental scheme and ranking analysis of remyelination
8. Supplementary figure legends

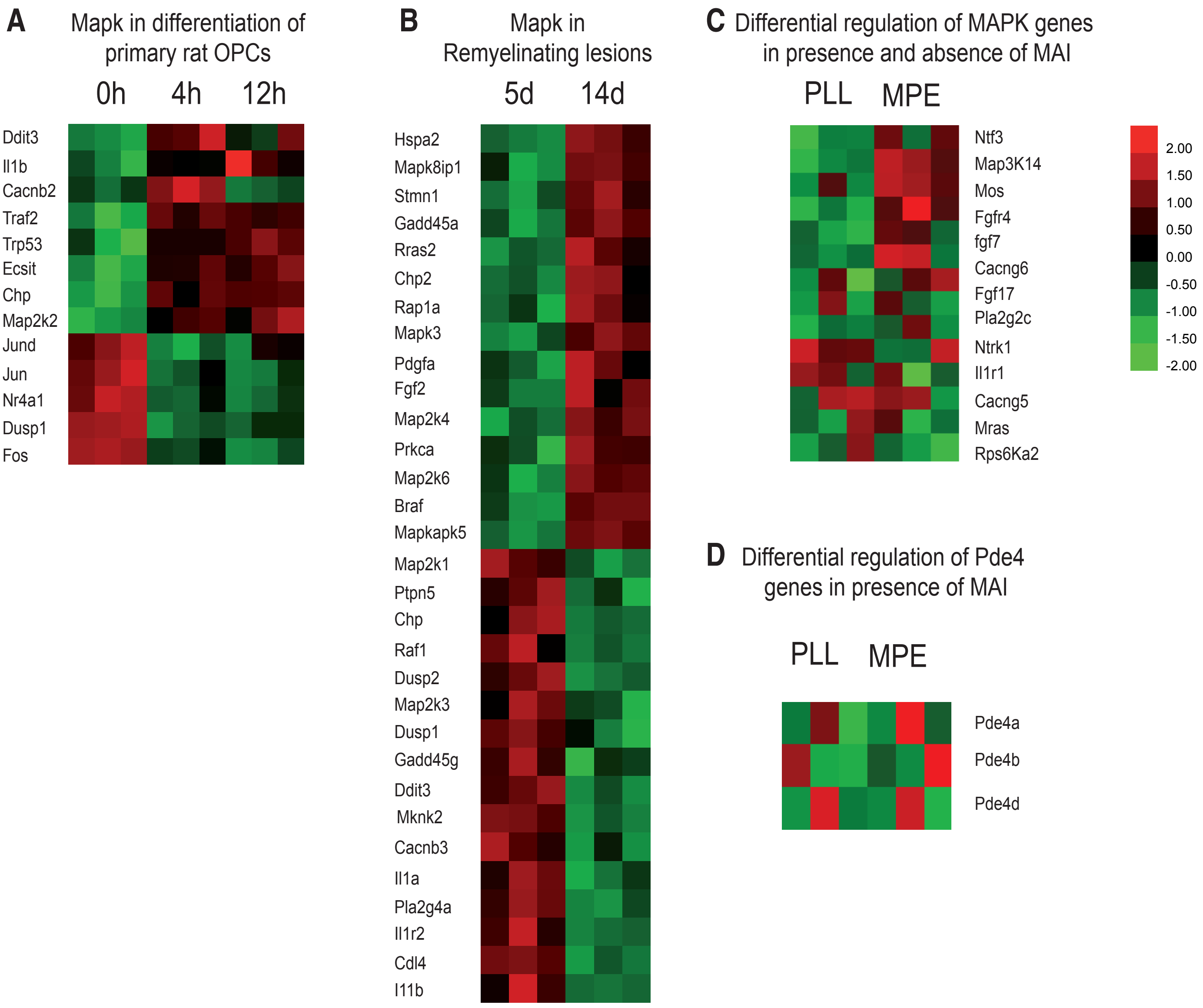
A
Mapk in differentiation of primary rat OPCs
0h
4h
12h
Ddit3
Il1b
Cacnb2
Traf2
Trp53
Ecsit
Chp
Map2k2
Jund
Jun
Nr4a1
Dusp1
Fos
B
Mapk in Remyelinating lesions
5d
14d
Hspa2
Mapk8ip1
Stmn1
Gadd45a
Rras2
Chp2
Rap1a
Mapk3
Pdgfa
Fgf2
Map2k4
Prkca
Map2k6
Braf
Mapkapk5
Map2k1
Ptpn5
Chp
Raf1
Dusp2
Map2k3
Dusp1
Gadd45g
Ddit3
Mknk2
Cacnb3
Il1a
Pla2g4a
Il1r2
Cdl4
I11b
C
Differential regulation of MAPK genes in presence and absence of MAI
PLL
MPE
Ntf3
Map3K14
Mos
Fgfr4
fgf7
Cacng6
Fgf17
Pla2g2c
Ntrk1
Il1r1
Cacng5
Mras
Rps6Ka2
2.00
1.50
1.00
0.50
0.00
-0.50
-1.00
-1.50
-2.00
D
Differential regulation of Pde4 genes in presence of MAI
PLL
MPE
Pde4a
Pde4b
Pde4d

Suppl Fig 1

A

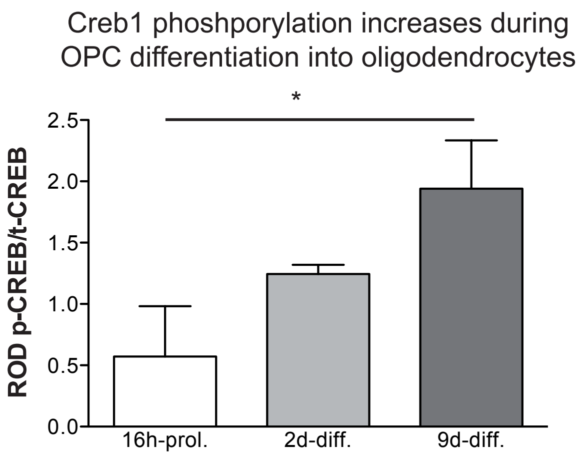
Creb1 phoshporylation increases during
OPC differentiation into oligodendrocytes
*
ROD p-CREB/t-CREB
2.5
2.0
1.5
1.0
0.5
0.0
16h-prol.
2d-diff.
9d-diff.

Suppl Fig 2

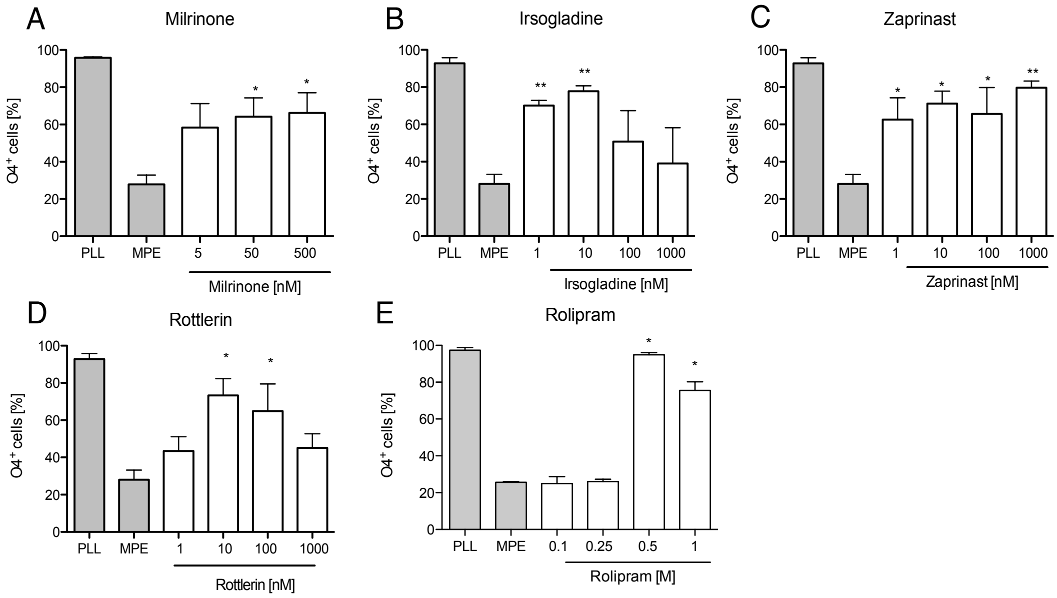
A
Milrinone
O4+ cells [%]
100
80
60
40
20
0
PLL
MPE
5
50
500
Milrinone [nM]
B
Irsogladine
1
10
100
1000
Irsogladine [nM]
C
Zaprinast
Zaprinast [nM]
D
Rottlerin
Rottlerin [nM]
E
Rolipram
0.1
0.25
0.5
Rolipram [M]

Suppl Fig 3

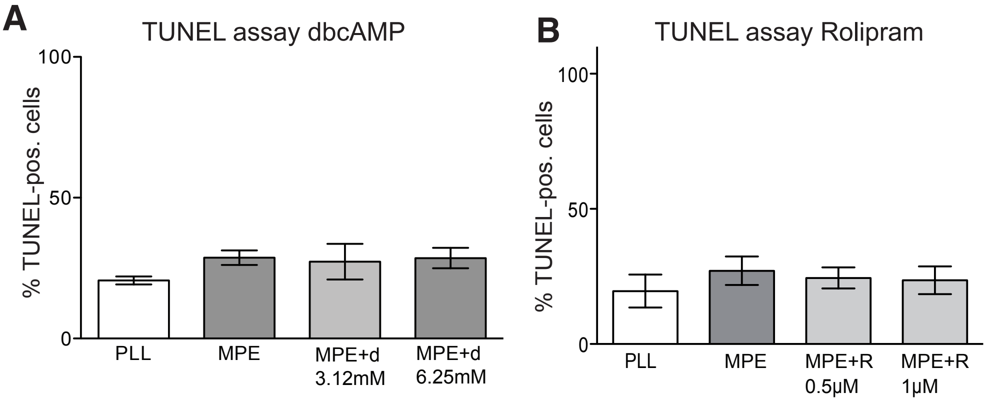
A
TUNEL assay dbcAMP
% TUNEL-pos. cells
100
50
0
PLL
MPE
MPE+d 3.12mM
MPE+d 6.25mM
B
TUNEL assay Rolipram
% TUNEL-pos. cells
100
50
0
PLL
MPE
MPE+R 0.5μM
MPE+R 1μM

Suppl Fig 4

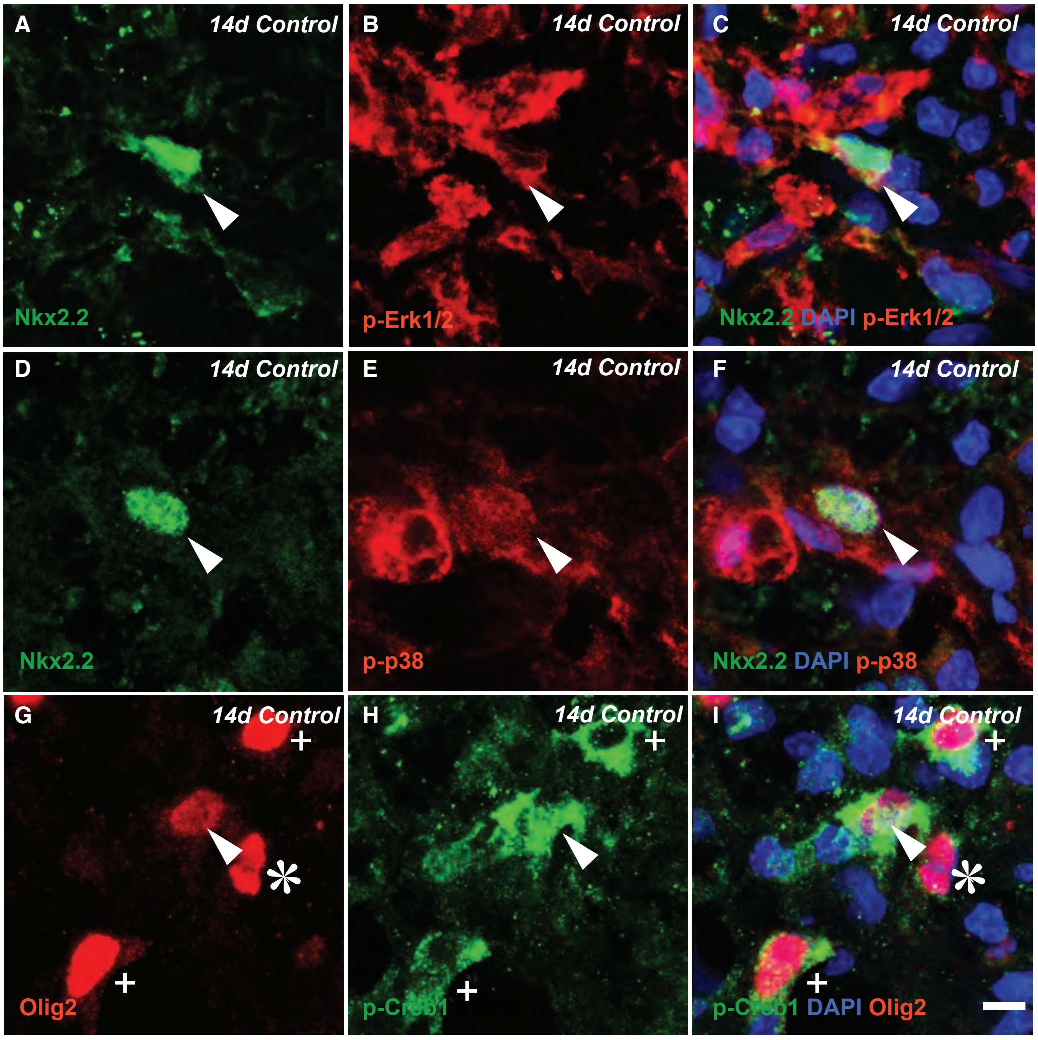
A
14d Control
Nkx2.2
B
14d Control
p-Erk1/2
C
14d Control
Nkx2.2 DAPI p-Erk1/2
D
14d Control
Nkx2.2
E
14d Control
p-p38
F
14d Control
Nkx2.2 DAPI p-p38
G
14d Control
Olig2
H
14d Control
p-Creb1
I
14d Control
p-Creb1 DAPI Olig2

Suppl Fig 5

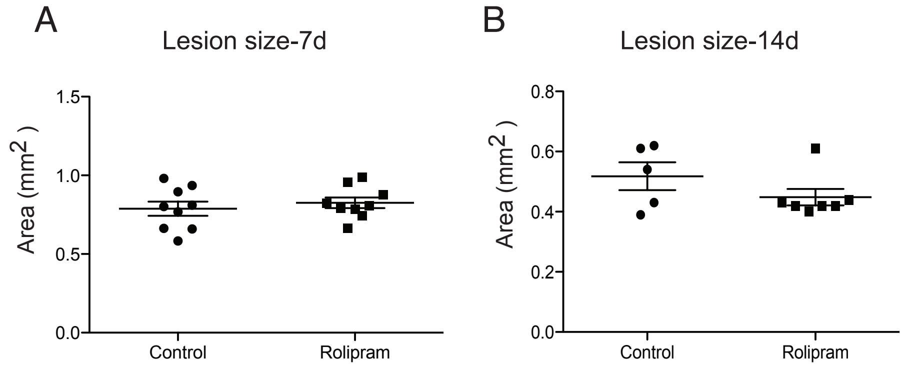
A
Lesion size-7d
1.5
1.0
0.5
0.0
Area (mm2)
Control
Rolipram
B
Lesion size-14d
0.8
0.6
0.4
0.2
0.0
Area (mm2)
Control
Rolipram

Suppl Fig 6

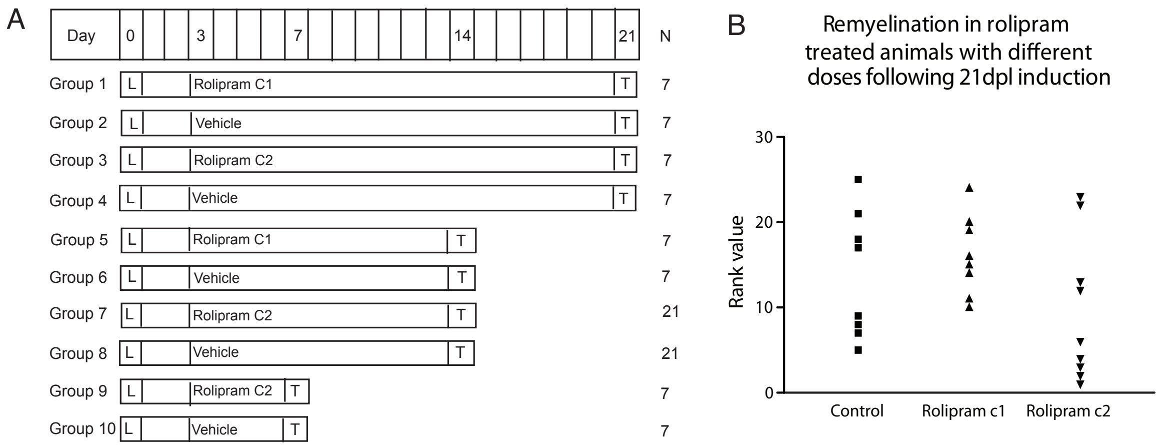
A
Day
0
3
7
14
21
N
Group 1
L
Rolipram C1
T
7
Group 2
L
Vehicle
T
7
Group 3
L
Rolipram C2
T
7
Group 4
L
Vehicle
T
7
Group 5
L
Rolipram C1
T
7
Group 6
L
Vehicle
T
7
Group 7
L
Rolipram C2
T
21
Group 8
L
Vehicle
T
21
Group 9
L
Rolipram C2
T
7
Group 10
L
Vehicle
T
7
B
Remyelination in rolipram treated animals with different doses following 21dpl induction
Rank value
30
20
10
0
Control
Rolipram c1
Rolipram c2

Suppl Fig 7

Supporting Information

Supplementary Figure Legends

Suppl. Fig. 1 Heat-map representation of some Mapk genes identified by microarray analysis.

A) Transcription profile of differentially expressed Mapk genes during early stages of oligodendrocyte differentiation (0h, 4h, 12h).

B) Differentially expressed Mapk gene expression in CCP lesions at 5 and 14 days post lesion induction (from Huang et al., 2011).

C,D) Microarray comparing differential Mapk gene expression in OPCs plated on PLL control substrates and myelin protein extracts (MPE) 4h following induction of differentiation. Mapk related genes (C), and Pde4 isoforoms (D)**.**

Bright red indicates the highest normalized intensity value, bright green the lowest, and black median values.

Suppl. Fig. 2 Creb1 activity increases during oligodendrocyte differentiation.

A) Creb1 activation at different time points of OPC differentiation was assessed by immunoprecipitation with antibodies against total Creb1 followed by Western blots with anti-p-Creb. ROD ratios of p-Creb1/t-Creb demonstrate a significant increase of Creb1 activity as the cells differentiate (n=3, ANOVA *P>0.05). Error bars indicate ± SE. (prol.=cells cultured under proliferating conditions, diff.=cells cultured under differentiating conditions)

Suppl. Fig. 3 Treatment with Pde4 inhibitors promote OPC differentiation in the presence of MAI.

A-E) Bar graph showing percentage of O4-positive cells following two days of differentiation on control substrate (PLL) or on MAI (MPE) in the presence or absence of various concentrations of Pde4 inhibitors. The drugs tested include milrinone, irsogladine, zaprinast, rottlerin, and rolipram (n ≥ 3; ANOVA: *P<0.05, **P<0.01; Dunnett's post-hoc test: *P<0.05, **P<0.01).

Suppl. Fig. 4 Quantification of apoptotic cells by TUNEL assay.

A, B) Bar graph showing the percentage of TUNEL positive cells. No difference was observed in the extent of apoptosis in OPCs cultured on PLL and on MPE in the presence and absence of (A) dbcAMP (d) and (B) rolipram (R) at different concentrations (n=3, ANOVA P>0.05).

Suppl. Fig. 5 Double immunohistochemistry conducted at the peak of the differentiation phase in remyelinating lesions (14 dpl) confirms expression of activated Erk1/2, p38Mapk, and Creb1 in OPCs.

A-C) Sections stained with antibodies against Nkx2.2 in combination with anti-p-Erk1/2. Arrowheads indicate the presence of active Erk1/2.

D-F) Sections stained with antibodies against Nkx2.2 in combination with anti-p-p38Mapk. Arrowheads indicate p-p38Mapk.

G-I) Sections of remyelinating lesions were co-stained with antibodies against Olig2 and p-Creb1. Activated Creb1 was highly expressed in Olig2 low-expressing (arrowheads) and Olig2 high-expressing cells (+); reduced levels were found in OPCs undergoing cell division (asterisks). Scale bar=50μm.

Suppl. Fig. 6 Rolipram treatment does not change the size of the lesion.

A) The lesion area was determined by using ImageJ software at day 7 days post lesion induction. No significant difference was observed between the groups. 7d control: n=9; 7d rolipram: n=9, t-test P>0.05, 7d control: 0.7886±0.04496; 7d rolipram 0.8259±0.03381

B) Following 14 dpl. no difference in lesion area was observed between the groups.; 14d control: n=5, 14d rolipram: n=7, t-test P>0.05, 14d control: 0.5180±0.04663, 14d rolipram: 0.4486±0.02729. Error bars indicate the ±SE.

Suppl. Fig. 7 Experimental scheme and ranking analysis of remyelination

A) Design of in vivo experiments.

B) Rank-based assessment of remyelination at 21 dpl indicates comparable levels between the groups. (Control=vehicle; rolipram c1=rolipram administration 0.5mg/kg/day; Rolipram c2=rolipram administration 2.64mg/kg/day; L=lesion induction, T=termination, N=number of animals).
